# Efficacy and safety of *Panax notoginseng* saponins (Xuesaitong) for patients with acute ischemic stroke: a systematic review and meta-analysis of randomized controlled trials

**DOI:** 10.3389/fphar.2023.1280559

**Published:** 2023-10-16

**Authors:** Xinyi Shi, Luda Feng, Yixuan Li, Mingzhen Qin, Tingting Li, Zixin Cheng, Xuebin Zhang, Congren Zhou, Sisong Cheng, Chi Zhang, Ying Gao

**Affiliations:** ^1^ Department of Neurology, Dongzhimen Hospital, Beijing University of Chinese Medicine, Beijing, China; ^2^ Dongfang Hospital, Beijing University of Chinese Medicine, Beijing, China; ^3^ Department of Psychiatry, Massachusetts General Hospital and Harvard Medical School, Charlestown, MA, United States; ^4^ Institute for Brain Disorders, Beijing University of Chinese Medicine, Beijing, China

**Keywords:** acute ischemic stroke, Xuesaitong, *Panax notoginseng* saponins, long-term functional outcomes, neurological deficits, systematic review, meta-analysis

## Abstract

**Background:** Stroke is the major cause of mortality and permanent disability and is associated with an astonishing economic burden worldwide. In the past few decades, accumulated evidence has indicated that Xuesaitong (XST) has therapeutic benefits in cases of acute ischemic stroke (AIS). Our study aimed to provide the best current body of evidence of the efficacy and safety of XST for patients with AIS.

**Methods:** This is a systematic review and meta-analysis of randomized controlled trials (RCTs). We searched eight electronic databases from inception to 17 July 2023 for relevant RCTs. The investigators independently screened trials, extracted data, and assessed the risk of bias. A meta-analysis was conducted using RevMan 5.3 and STATA 16.0 software.

**Results:** In total, 46 RCTs involving 7,957 patients were included. The results showed that XST improved the long-term functional outcomes with lower modified Rankin Scale (mRS) scores (MD = −0.67; 95% CI [−0.92 to −0.42]; *p* < 0.00001) and a higher proportion of functional independence (mRS ≤2) (RR = 1.08; 95% CI [1.05 to 1.12]; *p* < 0.00001). Low-quality evidence indicated that XST improved the activities of daily living (MD = 10.17; 95% CI [7.28 to 13.06]; *p* < 0.00001), improved the neurological impairment (MD = −3.39; 95% CI [−3.94 to −2.84]; *p* < 0.00001), and enhanced the total efficiency rate (RR = 1.19; 95% CI [1.15 to 1.23]; *p* < 0.00001). No significant difference was found in the all-cause mortality or incidence of adverse events between the XST and control groups. The certainty of evidence was estimated as moderate to very low.

**Conclusion:** Presently, the administration of XST within 14 days of AIS is associated with favorable long-term functional outcomes. In addition, XST can improve activities of daily living, alleviate neurological deficits, and has shown good tolerability. However, the current evidence is too weak, and the confidence of evidence synthesis was restricted by the high risk of bias. Given the insufficient evidence, appropriately sized and powered RCTs investigating the efficacy and safety of XST for patients with AIS are warranted.

**Systematic Review Registration:** https://www.crd.york.ac.uk/PROSPERO/display_record.php?RecordID=446208, CRD42023446208.

## 1 Introduction

Stroke is the major cause of mortality and permanent disability worldwide and is associated with a high lifetime risk (Feigin et al., 2017; GBD 2019 Diseases and Injuries Collaborators, 2020). The high incidence and disability of stroke lead to an astonishing economic burden annually (Wu et al., 2019a; Rajsic et al., 2019). Ischemic stroke accounts for 69.6% of incident strokes and 77.8% of prevalent strokes and is regarded as the most common stroke subtype (Wang et al., 2017b).

In select patients with non-minor acute ischemic stroke (AIS), intravenous thrombolysis within 4.5 h and mechanical thrombectomy initiated within 24 h of symptom onset could salvage the ischemic penumbra and improve functional outcomes (Mendelson and Prabhakaran, 2021). Despite the clear benefit within a specified time window, stroke thrombolysis is highly time-critical and has been limited by the unknown time from symptom onset (Meretoja et al., 2014), high cost, and limited medical level. Otherwise, short-term dual antiplatelet therapy administered within 24 h of symptom onset could reduce the risk of stroke in patients with minor AIS (Wang et al., 2013). However, the side effects associated with antiplatelet agents, including damage to the liver and kidney, gastrointestinal injuries (Nema and Kato, 2010), and an increased risk of moderate to severe bleeding (Bhatia et al., 2021), should be taken into account; limitations, including aspirin resistance (AR) (Hankey and Eikelboom, 2006) and CYP2C19 genetic variants (Wang et al., 2016), restrict the clinical applications. Given the clinical dilemma, it is imperative to optimize stroke medication by developing and confirming safer and more effective therapies benefiting more patients with AIS.

Research on neuroprotective agents for AIS has been ongoing but has frequently failed to achieve the anticipated benefits in several clinical trials (Paul and Candelario-Jalil, 2021). *Panax notoginseng* (Burk.) F.H. Chen, also called Sanqi or Tianqi in Chinese, is an extremely valued herbal medicine in Asia. *Panax notoginseng* saponins (PNSs), the bioactive ingredients of *P. notoginseng*, consist of multiple active components and include five main bioactive ingredients accounting for 90% of the total PNSs: notoginsenoside R1, ginsenoside Rg1, ginsenoside Rb1, ginsenoside Rd, and ginsenoside Re (Qu et al., 2020). PNSs have been used for the clinical treatment of AIS since antiquity and have exerted obvious anti-inflammatory effects on atherosclerosis-related cardiac–cerebral vascular disease (Wan et al., 2009; Wang et al., 2011b). The pathophysiology of cerebral ischemic injury is correlated with a rapid cascade of energy failure, excitotoxicity, oxidative stress, nitrative stress, and inflammatory responses (Dirnagl et al., 1999; Chamorro et al., 2016). Neuroinflammation is considered a potential treatment target for such complex and dynamic processes (Jayaraj et al., 2019). PNS and notoginsenoside R1 exhibit versatile biological activities, including anti-inflammatory activity (Shi et al., 2017), antioxidant capacity (Zhang et al., 2019), alleviation of blood–brain barrier (BBB) disruption (Wu et al., 2019b), antiapoptosis (Chen et al., 2011), and endothelial cell protection (Hu et al., 2018). Xuesaitong (XST), one of the most commonly used medicinal products of PNS-related preparations for treating AIS, was licensed for ischemic stroke by the National Medical Products Administration in China in 1999. The experimental studies indicated that the neuroprotective mechanisms of XST included antioxidation (Zhou et al., 2014) and antiapoptosis (Li et al., 2009), and significant reduction was found in the infarct volume and neurologic impairment in mice models with middle cerebral artery occlusion when XST was administered during the acute phase of ischemic stroke (Li et al., 2019). The reported quality control (Yang et al., 2017) and the previous post-marketing surveillance study (He et al., 2020) provided some evidence of the effectiveness and safety of XST for clinical applications. Consequently, XST, composed of multiple active components, might produce clinically effective neuroprotection for the treatment of AIS.

In the past few decades, accumulated evidence has indicated that XST has therapeutic benefits in cases of AIS. Recent meta-analyses of randomized controlled trials (RCTs) (Zhang et al., 2015; Geng et al., 2022) have evaluated the efficacy of XST for patients with AIS; however, the findings were discordant and inconclusive. Previous meta-analyses analyzed the efficacy of XST oral preparation or XST injection. However, the safety of XST and whether XST improves long-term functional outcomes and reduces all-cause mortality remain unknown, which has probably led to inadequate comprehension of the clinical benefits of XST for patients with AIS. Moreover, it is worth mentioning that the latest multicenter, double-blind, placebo-controlled randomized clinical trial conducted by our team provided strong new evidence of XST efficacy and safety in patients with AIS (Wu et al., 2023). To summarize and provide the best current evidence regarding the benefits and harm of XST treatment for patients with AIS, we conducted this systematic review to examine the efficacy and safety of XST on AIS without differentiating dosage forms.

## 2 Materials and methods

We performed and reported this systematic review and meta-analysis based on the Preferred Reporting Items for Systematic Reviews and Meta-analyses (PRISMA) 2020 statement (Page et al., 2021). The protocol was already registered in the International Prospective Register of Systematic Reviews (PROSPERO) (CRD42023446208).

### 2.1 Search strategy

A comprehensive search was conducted to identify published studies of RCTs indexed in PubMed, Embase, the Cochrane Library, Web of Science, the Chinese National Knowledge Infrastructure (CNKI), the Chinese Science and Technology Journals Database (VIP), the Wanfang Database, and SinoMed without language limitations from their respective inception dates to 17 July 2023. The Medical Subject Heading (MeSH) terms and free-text keywords were utilized. We also searched the registered clinical trials, dissertations, and gray literature. In addition, a secondary manual search was conducted according to the references of the included articles. The detailed search strategies are presented in the Supplementary Material.

### 2.2 Eligibility criteria

#### 2.2.1 Inclusion criteria

(1) Types of studies: RCTs evaluating the efficacy and safety of XST for patients with AIS were included.
(2) Type of participants: Participants diagnosed with AIS (within 14 days of symptom onset), defined in accordance with the Fourth National Conference on Cerebrovascular Disease by the Chinese Medical Association in 1995, without sex, age, or race restrictions.
(3) Type of interventions: Intervention groups were treated with XST injection or XST oral preparations, regardless of the treatment duration and dosage. Control groups were treated with a placebo, conventional treatment, neuroprotective agents, or other cointerventions.
(4) Type of outcomes: The primary outcome was the improvement of long-term functional outcomes, assessed by the modified Rankin Scale (mRS) score or Glasgow Outcome Scale (GOS) grades. The secondary outcomes were all-cause mortality, activities of daily living assessed by the Barthel Index (BI) score, neurological impairments assessed by clinical scales including the National Institute of Health Stroke Scale (NIHSS), European Stroke Scale (ESS), Canadian Neurological Scale (CNS), Scandinavian Stroke Scale (SSS), Modified Edinburgh–Scandinavian Stroke Scale (MESSS), and other related scales, the total efficiency rate, and blood rheology indicators including whole blood high-cut viscosity (HBV), whole blood low-cut viscosity (LBV), fibrinogen (FIB), plasma viscosity (PV), hematocrit (Hct), and other related indicators. Safety outcomes were measured as the occurrence of XST-induced adverse events.

#### 2.2.2 Exclusion criteria

RCTs with crossover and N-of-1 designs were excluded.

### 2.3 Study selection

After removing duplicate studies in all records retrieved, two reviewers (XS and ZC) screened the titles and abstracts independently, and three reviewers (YL, ZC, and CZ) independently selected the articles meeting eligibility criteria through full-text search. Disagreements were discussed, and a third author (YG) was contacted to arbitrate.

### 2.4 Data extraction

Reviewers, in pairs (YL and ZC, SC, and CZ), independently performed the data extraction using a preformulated data collection form as follows: 1) information from the included studies concerning the authors, publication year, and title; 2) patient characteristics, including the number of participating sites, sample sizes, age, sex, and onset time; 3) intervention details, including dosage form, dosage, frequency, duration, and combination treatment; and 4) outcomes.

### 2.5 Assessment of risk of bias

Two reviewers (TL and MQ) independently evaluated and cross-checked the risk of bias for eligible RCTs according to the Cochrane risk of bias tool 2.0. We evaluated five items as follows: “randomization process,” “deviations from intended interventions,” “missing outcome data,” “outcome measurements,” and “selective reporting.” Finally, each item was classified into “low risk of bias,” “some concerns,” and “high risk of bias.” Any disagreement was resolved by discussion and in consultation with a third author (CZ).

### 2.6 Data synthesis and analysis

Statistical analyses were conducted using RevMan 5.3 software and STATA 16.0. The results were expressed herein as the relative risk (RR) for dichotomous variables, whereas the mean difference (MD) was used for continuous data. The effect estimates were measured with a 95% confidence interval (CI), and *p* < 0.05 was considered to be statistically significant.

Statistical heterogeneity among studies was evaluated using the I-square (I^2^) statistic test. Data with I^2^ ≤ 25% were defined as insignificant heterogeneity, and we selected a fixed-effects model. When the baseline characteristics were acceptable and statistical heterogeneity was comparable (I^2^ > 25%), a random-effects model was adopted.

When I^2^ > 25%, we conducted the sensitivity analyses, iteratively omitting each study one at a time. Furthermore, subgroup analyses were performed regarding the duration of treatment and dosage form. If the statistical heterogeneity could be successfully explained by the sensitivity analysis or the subgroup analysis (I^2^ ≤ 25%), we applied a fixed-effects model. If not, a random-effects model was adopted. Considering that I^2^ could be biased in small meta-analyses, we adopted a random-effects model for such analysis (von Hippel, 2015).

### 2.7 Publication bias

Potential publication bias was detected by visually inspecting the funnel plot symmetry, and we conducted Begg’s statistical tests for ≥20 included studies and Egger’s statistical tests for <20 included studies.

### 2.8 Quality of evidence

According to the Grading of Recommendations, Assessment, Development, and Evaluation (GRADE) (Balshem et al., 2011), two independent reviewers (XS and LF) evaluated the quality of the evidence derived from the meta-analysis result. We rated the evidence as “high,” “moderate,” “low,” or “very low.” Disagreements regarding upgrades or downgrades were resolved by a third reviewer (YG).

## 3 Results

### 3.1 Literature search

**FIGURE 1:**
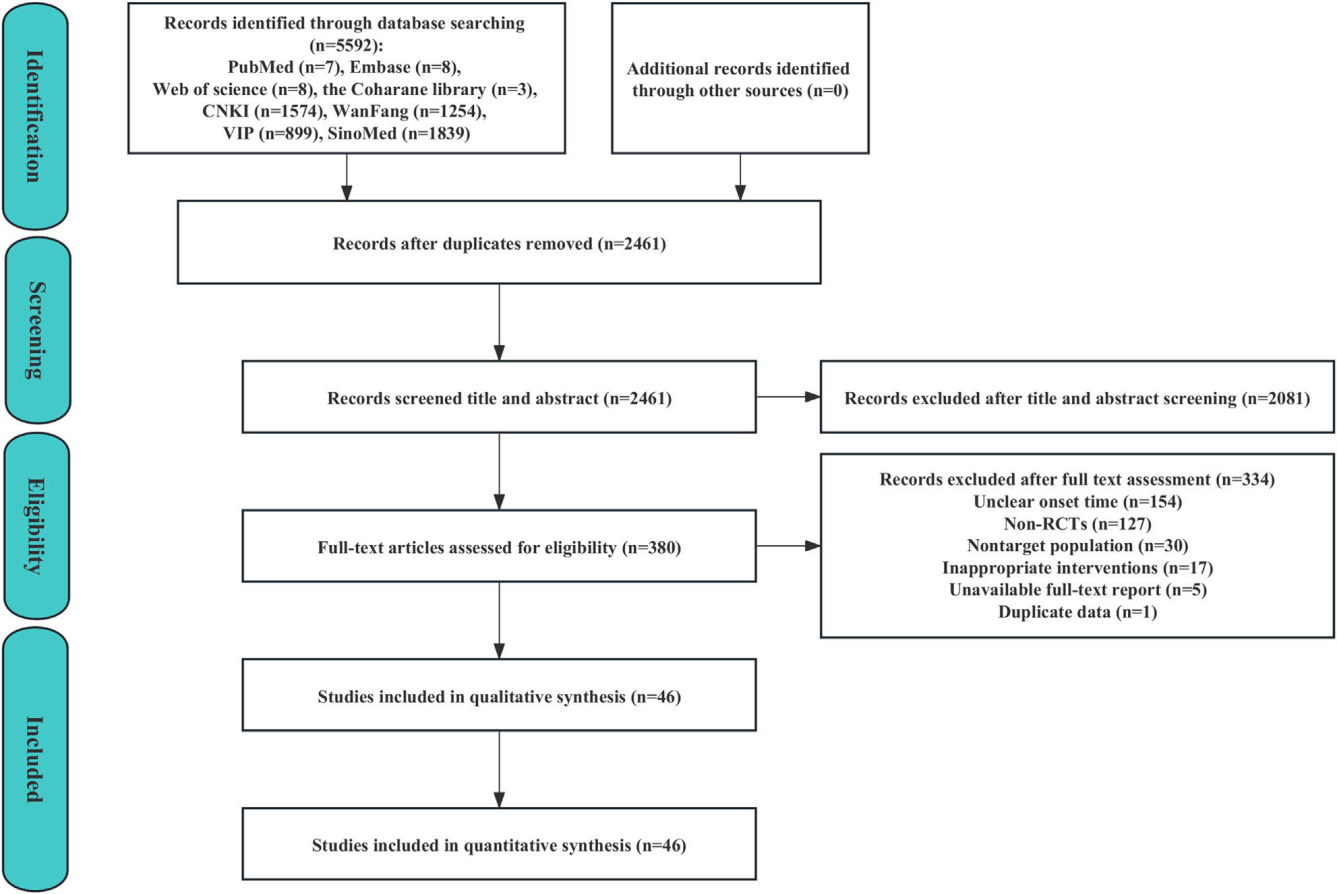
Flow diagram of study selection.

The electronic search identified 5,592 potentially relevant publications. Of these, 3,131 duplicates were removed, and 2,081 were excluded after screening the titles and abstracts. Of the remaining 380 articles that were subjected to a full-text review, 334 ineligible studies were excluded for the following reasons: unclear onset time (154 studies), non-RCTs (127 studies), non-target population (30 studies), inappropriate interventions (17 studies), unavailable full-text report (5 studies), and duplicate data (1 study). Ultimately, a total of 46 studies were eventually included in the quantitative analysis; the PRISMA flow diagram is shown in Figure 1.

### 3.2 Characteristics of the included studies

Overall, the 46 eligible RCTs were published between 2011 and 2023 and involved 7,957 participants, with 3,983 classified in the experimental groups and 3,974 in the control groups. The sample size ranged from 50 to 3,072, and most of the participants were middle-aged or elderly, with a mean age ranging from 54.5 to 72.5 years. In all, 45 RCTs were single-center trials, and 1 RCT was a multicenter trial. Regarding the dosage form, 43 studies used XST injections, whereas only 3 studies used XST oral preparations. We summarize the characteristics of the included studies in Table 1.

**TABLE 1:** Characteristics of the studies included in this meta-analysis.

Study ID	Onset time	Sample size		Sex (M/F)		Average age		Dosage form	Dosage	Combination treatment	Duration	Outcome
		T	C	T	C	T	C					
Bu (2016)	1–3d	30	30	16/14	18/12	60.4 ± 11.3	58.6 ± 10.9	Injection	400 mg iv qd	NAT, CT	28d	②
Cao et al. (2016)	<48 h	40	40	22/18	23/17	67.8 ± 7.9	67.4 ± 7.5	Injection	400 mg iv qd	NAT, CT	14d	②③⑤
Deng (2018)	1–4d	25	25	10/15	13/12	57.4 ± 8.2	59.4 ± 7.3	Capsule	50 mg po tid	CT	14d	③
Fan et al. (2019)	<48 h	150	150	95/55	88/52	67.16 ± 6.53	67.77 ± 6.48	Injection	200 mg iv qd	CT	14d	②③
Fu et al. (2011)	<48 h	64	58	32/30	30/28	54.5 ± 5.6	56.3 ± 4.5	Injection	400 mg iv qd	CT	14d	②③
Gao (2017)	1–3d	49	49	25/24	24/25	58.4 ± 7.2	58.7 ± 7.6	Injection	400 mg iv qd	NAT, CT	28d	②
Guo (2017)	24–72 h	75	75	40/35	42/33	70 ± 5	71 ± 5	Injection	500 mg iv qd	CT	14d	②③④
Han et al. (2014)	<3d	41	40	26/15	22/18	72.53 ± 9.24	71.45 ± 8.67	Injection	500 mg iv qd	CT	14d	②③
Hu et al. (2017)	<72 h	56	52	30/26	27/25	68.28 ± 7.26	67.51 ± 8.33	Injection	400 mg iv qd	NAT, CT	28d	②③⑤
Huang (2016)	<48 h	62	62	41/21	41/21	59.2 ± 11.1	58.7 ± 11.7	Injection	400 mg iv qd	CT	14d	②⑤
Huang et al. (2022)	<24 h	40	40	24/16	25/15	69.4 ± 3.2	69.7 ± 2.8	Injection	400 mg iv qd	NAT, CT	14d	②③⑥
Li et al. (2015)	<48 h	62	62	40/22	38/24	59.2 ± 7.8	59.8 ± 7.4	Injection	400 mg iv qd	CT	14d	②③⑤
Li and Chang (2016)	<24 h	48	48	26/22	28/20	58.9 ± 8.5	59.5 ± 9.3	Injection	400 mg iv qd	NAT, CT	28d	②③④
Li et al. (2016)	<36 h	60	60	38/22	35/25	66.4 ± 7.1	67.0 ± 5.8	Injection	400 mg iv qd	CT	14d	②
Li (2017)	1–13d	74	74	38/36	37/37	72 ± 8	73 ± 8	Injection	400 mg iv qd	NAT, CT	14d	②
Li and Liu (2020)	<36 h	34	34	20/14	19/15	57.92 ± 2.52	57.86 ± 2.85	Injection	4–8 mL iv qd	CT	28d	②③
Liu (2017)	1–3d	120	120	65/55	64/56	60.23 ± 4.75	60.56 ± 4.48	Injection	400 mg iv qd	NAT, CT	28d	②③
Liu et al. (2019)	<30 h	48	48	31/17	29/19	68.32 ± 5.63	68.59 ± 5.47	Injection	400 mg iv qd	NAT, CT	14d	②③④⑥
Luo et al. (2011)	<72 h	30	30	17/13	18/12	61.37 ± 2.24	62.35 ± 8.76	Injection	400 mg ivqd	CT	14d	②③⑥
Ouyang and Zhang (2022)	<7 h	48	48	24/24	25/23	61.25 ± 5.35	61.13 ± 5.25	Injection	500 mg iv qd	NAT, CT	14d	②⑤
Ping et al. (2022)	<24 h	46	46	28/18	30/16	66.62 ± 4.10	66.55 ± 4.05	Injection	200 mg iv qd	NAT, CT	14d	③⑤⑥
Shen and Zhang (2017)	4–22 h	50	50	28/22	29/21	64.19 ± 4.01	64.32 ± 4.24	Injection	400 mg iv qd	CT	14d	⑤⑥
Song et al. (2022)	<5 h	41	41	25/16	23/18	60.27 ± 0.02	60.13 ± 1.35	Injection	200–400 mg iv qd	NAT, CT	14d	②③⑥
Wang (2015)	2–48 h	43	43	-	-	-	-	Injection	400 mg iv qd	CT	14d	②③④⑤
Wang and Ning (2018)	1–3d	43	43	25/18	24/19	62.21 ± 4.33	61.30 ± 5.23	Injection	400 mg iv qd	CT	14d	②③④
Wang et al. (2020)	<72 h	53	53	29/24	31/22	56.13 ± 6.29	56.27 ± 6.24	Injection	200 mg iv qd	NAT, CT	14d	②③⑤⑥
Wang et al. (2021)	<30 h	51	51	27/24	26/25	61.46 ± 5.28	62.26 ± 4.64	Injection	400 mg iv qd	CT	14d	②③④⑤⑥
Wang (2022)	<24 h	39	39	23/16	24/15	62.5 ± 4.6	62.4 ± 4.5	Injection	400 mg iv qd	CT	28d	②③④
Wu (2020)	<4d	34	34	20/14	18/16	56.1 ± 4.8	55.3 ± 4.3	Injection	400 mg iv qd	NAT, CT	28d	②⑤⑥
Wu et al. (2023)	<14d	1,535	1,537	957/530	1,025/454	62	62	Capsule	120 mg po bid	Placebo, CT	90d	①③④⑥
Xiao et al. (2019)	<48 h	65	65	42/23	40/25	55.16 ± 10.92	55.37 ± 10.49	Injection	2 mL Iv qd	CT	14d	②⑤
Xiao et al. (2020)	<48 h	39	39	21/18	23/16	58.72 ± 7.18	58.06 ± 8.27	Injection	400 mg iv qd	CT	14d	②⑤
Xu (2021)	<5 h	90	90	45/45	46/44	72.46 ± 10.31	71.34 ± 10.29	Injection	400 mg iv qd	CT	14d	②③④
Xue (2018)	<49 h	36	36	19/17	20/16	58.18 ± 6.52	57.89 ± 6.85	Injection	400 mg iv qd	CT	28d	②③⑤⑥
Yan et al. (2023)	<36 h	51	51	26/25	28/23	72.23 ± 4.72	72.15 ± 4.61	Injection	200–400 mg iv qd	CT	7d	②⑤⑥
Yang (2021)	<24 h	32	32	18/14	16/16	71.11 ± 1.95	72.34 ± 270	Injection	200–400 mg iv qd	NAT, CT	14d	②
Ye et al. (2018)	<24 h	43	43	22/21	23/20	58.56 ± 5.61	58.19 ± 5.43	Injection	100 mg iv qd	CT	14d	③⑤
Zhang (2016)	<72 h	50	50	-	-	-	-	Capsule	120 mg po bid	CT	28d	②③
Zhang (2018)	24–72 h	46	46	26/20	25/21	69.36 ± 6.72	68.94 ± 6.15	Injection	500 mg iv qd	CT	14d	②③④
Zhang (2019)	<48 h	29	29	19/10	18/11	54.3 ± 7.6	54.7 ± 7.1	Injection	400 mg iv qd	CT	14d	②⑥
Zhang et al. (2020)	<3d	58	58	37/21	39/19	59.24 ± 5.66	63.45 ± 4.21	Injection	500 mg iv qd	CT	14d	②③
Zhang et al. (2022)	<24 h	96	96	49/47	47/49	60.98 ± 3.16	61.32 ± 2.64	Injection	100 mg iv qd	CT	28d	②③④
Zhao (2022)	<4.5 h	90	90	47/43	48/42	55.71 ± 6.36	54.79 ± 6.15	Injection	250 mg iv qd	NAT, CT	14d	②③⑥
Zhong and Deng (2017)	1–5 h	42	42	31/11	30/12	61.85 ± 1.49	62.15 ± 1.52	Injection	400 mg iv qd	NAT, CT	28d	③④
Zhou et al. (2015)	24h–72 h	65	65	35/30	34/31	67.30 ± 5.19	67.21 ± 5.23	Injection	500 mg iv qd	CT	14d	②③④
Zhu et al. (2016)	<25 h	60	60	-	-	-	-	Injection	400 mg iv qd	NAT, CT	14d	②③⑥

NAT, neuroprotective agent treatment; CT, conventional treatment; d, day; h, hour; ①, long-term functional outcomes and reduction of all-cause mortality; ②, total efficiency rate; ③, improvement in neurological impairment; ④, improvement in activities of daily living; ⑤, blood rheology indicators; ⑥, adverse events.

### 3.3 Risk of bias assessment

**FIGURE 2:**
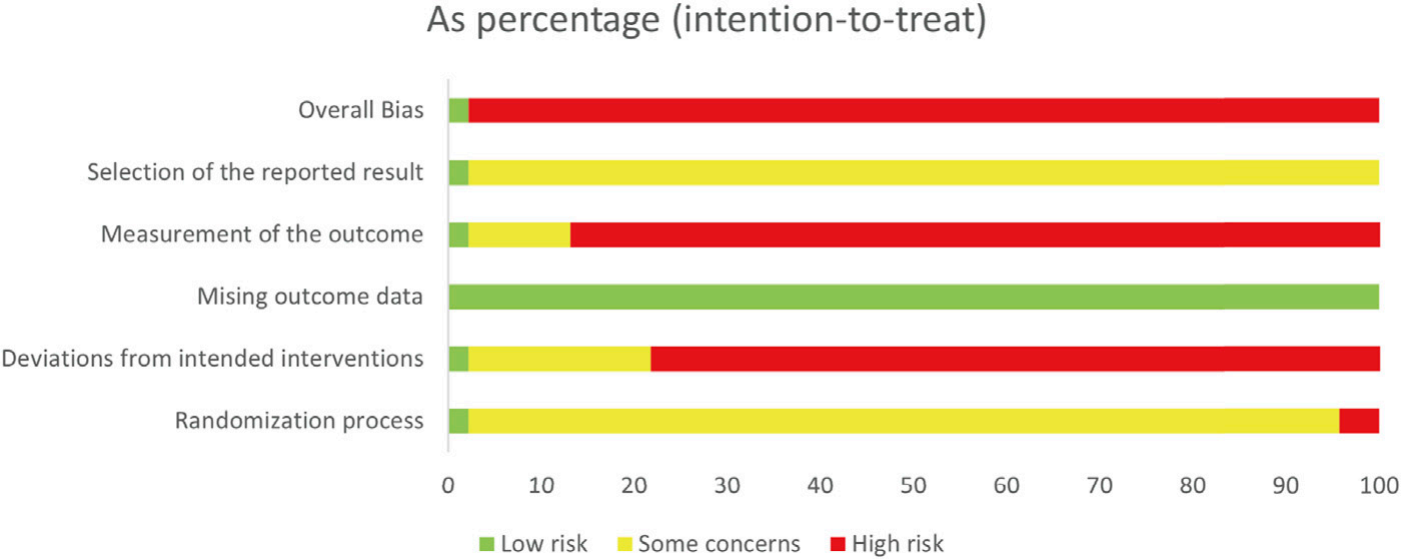
Risk of bias graph for each study.

We identified the overall bias as “low risk of bias” in one study (Wu et al., 2023) and judged the remaining 45 studies to have a “high risk of bias”, indicating the poor quality of the selected RCTs. The results of the assessment of bias risk are presented in Figure 2 and Supplementary Figure S1.

We identified the “randomization process” as having a “high risk of bias” for the inappropriate methods of allocation concealment in two studies. On the contrary, we identified one study as having a “low risk of bias” because the allocation sequence was stored by researchers who were not involved in the observation. Regarding the risk of bias due to the “deviations from the intended interventions”, we rated 36 studies as having a “high risk of bias” because they did not report blinding and the per-protocol principle was used in analyses. Conversely, we judged one study as having a “low risk of bias” for its double-blind study design and appropriate analyses such as intention to treat. In addition, we judged all studies as having a “low risk of bias” in the case of the “missing outcome data”. Because no reported loss to follow-up was detected, or we found negligible losses to follow-up, the missing data were balanced between the experimental group and control group. We identified the “outcome measurements” as having a “high risk of bias” in 40 studies, considering that the total efficiency rate is the composite endpoint. In contrast, we rated one study as having a “low risk of bias” for its objective outcome and blinded outcome assessors. With regard to the risk of bias due to the “selective reporting”, one study was identified as having a “low risk of bias” for its transparent report of the observations planned, while 45 studies were rated as having “some concerns” for the lack of relevant reporting.

### 3.4 Efficacy outcomes

#### 3.4.1 Long-term functional outcomes

**FIGURE 3:**
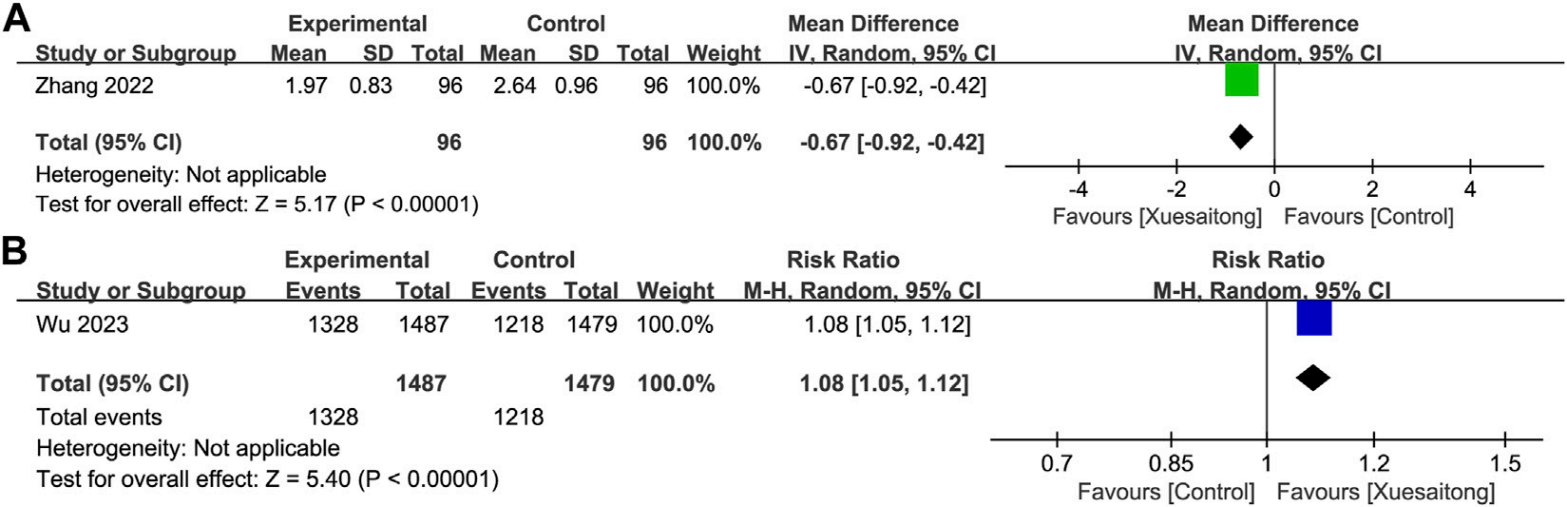
Forest plot for the effect of Xuesaitong on long-term functional outcomes by different outcomes. **(A)** By the grading of the modified Rankin Scale. **(B)** By the proportion of the modified Rankin Scale grade less than 3.

For the long-term functional outcomes, two studies (Zhang et al., 2022; Wu et al., 2023) comprising a total of 3,158 participants reported the grading or the proportion as per the mRS, and we were unable to synthesize the data. Patients in the XST group were more likely to have better long-term functional outcomes with lower mRS scores (MD = −0.67; 95% CI [−0.92 to −0.42]; *p* < 0.00001) or a higher proportion of functional independence (mRS ≤2) (RR = 1.08; 95% CI [1.05 to 1.12]; *p* < 0.00001) (Figure 3).

### 3.5 Secondary outcomes

#### 3.5.1 Reduction of all-cause mortality

**FIGURE 4:**
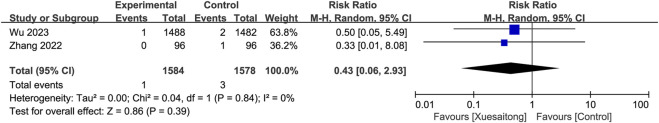
Forest plot for the effect of Xuesaitong on all-cause mortality.

Two studies (Zhang et al., 2022; Wu et al., 2023) containing 3,162 cases reported all-cause mortality, whereas there was no significant difference between the XST group and the control group (RR = 0.43; 95% CI [0.06 to 2.93]; *p* = 0.39; I^2^ = 0%) (Figure 4). As such, no evidence was found to indicate that XST could reduce the all-cause mortality of AIS.

#### 3.5.2 Improvement in activities of daily living

**FIGURE 5:**
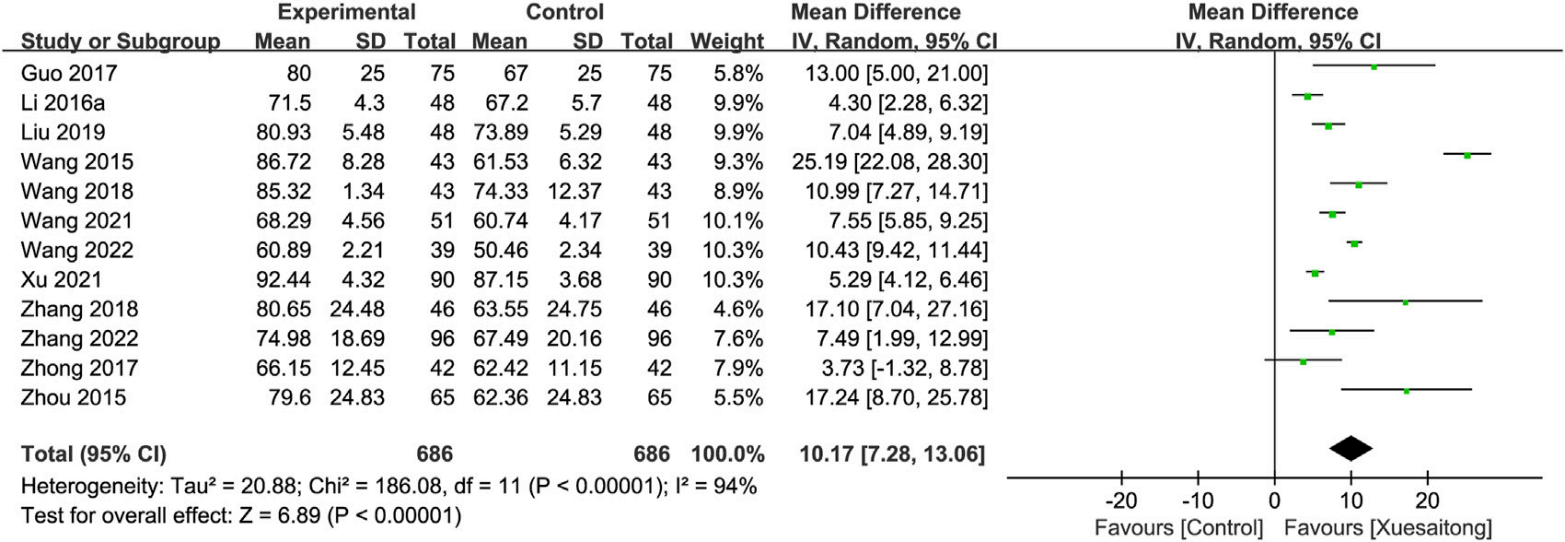
Forest plot for the effect of Xuesaitong on the Barthel Index score.

In all, 13 studies (Wang, 2015; Zhou et al., 2015; Li et al., 2016; Guo, 2017; Zhong and Deng, 2017; Wang and Ning, 2018; Zhang, 2018; Liu et al., 2019; Wang et al., 2021; Xu, 2021; Wang, 2022; Zhang et al., 2022; Wu et al., 2023) comprising 1,372 participants used the BI score; however, one of the studies (Wu et al., 2023) reported data on the BI score change from baseline to 90 days, and we were unable to synthesize this study. The pooled data of the other 12 studies clarified that XST improved the BI score (MD = 10.17; 95% CI [7.28 to 13.06]; *p* < 0.00001) (Figure 5). In view of the significant heterogeneity in the meta-analysis of the BI score (I^2^ = 94%, *p* < 0.00001), a random-effects model was used. Further sensitivity analysis showed that statistical heterogeneity was not significantly reduced when we excluded a single study in sequence. We performed subgroup analyses by the duration of treatment (14 days, MD = 12.40; 95% CI [7.85 to 16.95]; *p* < 0.00001; 28 days, MD = 6.66; 95% CI [2.42 to 10.90]; *p* < 0.00001) (Supplementary Figure S2) and by the combination treatment (conventional treatment, MD = 12.17; 95% CI [8.51 to 15.84]; *p* < 0.00001; neuroprotective agents plus conventional treatment, MD = 5.36; 95% CI [3.23 to 7.50]; *p* < 0.00001) (Supplementary Figure S3).

#### 3.5.3 Improvement in neurological impairment

**FIGURE 6:**
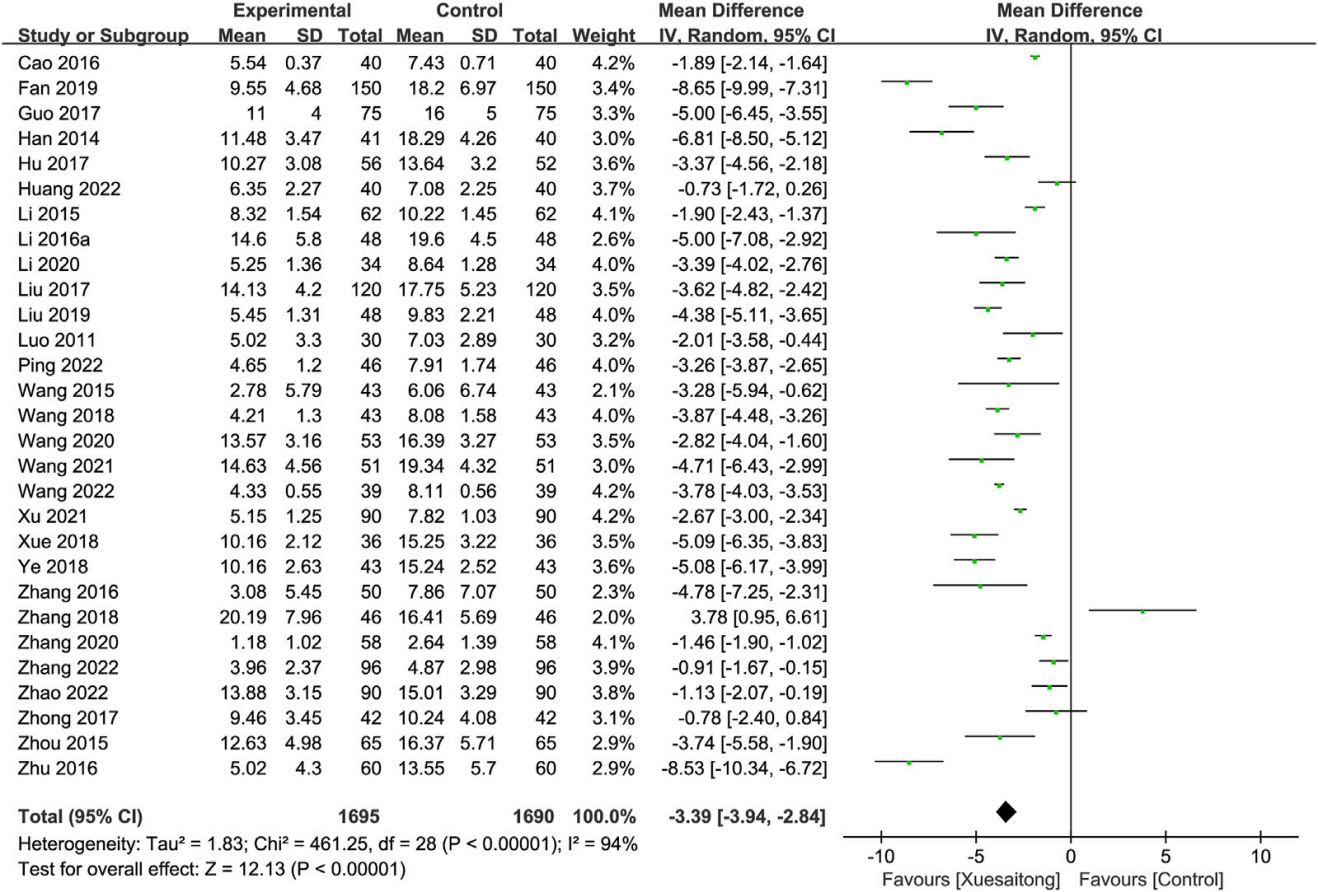
Forest plot for the effect of Xuesaitong on the National Institute of Health Stroke Scale score.

Regarding the improvement in neurological impairment, 30 studies (Luo et al., 2011; Han et al., 2014; Li et al., 2015; Wang, 2015; Zhou et al., 2015; Cao et al., 2016; Li et al., 2016; Zhang, 2016; Zhu et al., 2016; Guo, 2017; Hu et al., 2017; Liu, 2017; Zhong and Deng, 2017; Wang and Ning, 2018; Xue, 2018; Ye et al., 2018; Zhang, 2018; Fan et al., 2019; Liu et al., 2019; Li and Liu, 2020; Wang et al., 2020; Zhang et al., 2020; Wang et al., 2021; Xu, 2021; Huang et al., 2022; Ping et al., 2022; Wang, 2022; Zhang et al., 2022; Zhao, 2022; Wu et al., 2023) containing 3,385 cases reported the grading according to the NIHSS score, and we excluded one study (Wu et al., 2023) that reported data on the NIHSS score change from baseline to 90 days. The outcome indicated that XST reduced the NIHSS score (MD = −3.39; 95% CI [−3.94 to −2.84]; *p* < 0.00001), and a random-effects model was applied due to the high heterogeneity (I^2^ = 94%, *p* < 0.00001) (Figure 6). Sensitivity analysis indicated that the statistical heterogeneity was not significantly reduced through the sequential removal of any study (Supplementary Figure S4). Subgroup analyses were then performed, respectively, by the duration of treatment (14 days, MD = −3.42; 95% CI [−4.12 to −2.73]; *p* < 0.00001; 28 days, MD = −3.30; 95% CI [−4.19 to −2.42]; *p* < 0.00001) (Supplementary Figure S5), by the dosage form (XST injection, MD = −3.36; 95% CI [−3.91 to −2.80]; *p* < 0.00001; XST oral preparation, MD = −4.78; 95% CI [−7.25 to −2.31]; *p* = 0.0002) (Supplementary Figure S6), and by the combination treatment (conventional treatment, MD = −3.58; 95% CI [−4.30 to −2.86]; *p* < 0.00001; neuroprotective agents plus conventional treatment, MD = −3.10; 95% CI [−4.03 to −2.18]; *p* < 0.00001) (Supplementary Figure S7).

We also analyzed one study (Deng, 2018) containing 50 participants, the data of which showed that XST reduced the ESS score (MD = 11.85; 95% CI [2.07 to 21.63]; *p* = 0.02) (Supplementary Figure S8).

#### 3.5.4 Total efficiency rate

A total of 40 studies (Fu et al., 2011; Luo et al., 2011; Han et al., 2014; Li et al., 2015; Wang, 2015; Zhou et al., 2015; Bu, 2016; Cao et al., 2016; Huang, 2016; Li and Chang, 2016; Li et al., 2016; Zhang, 2016; Zhu et al., 2016; Gao, 2017; Guo, 2017; Hu et al., 2017; Li, 2017; Liu, 2017; Wang and Ning, 2018; Xue, 2018; Zhang, 2018; Fan et al., 2019; Liu et al., 2019; Xiao et al., 2019; Zhang, 2019; Li and Liu, 2020; Wang et al., 2020; Wu, 2020; Xiao et al., 2020; Zhang et al., 2020; Wang et al., 2021; Xu, 2021; Yang, 2021; Huang et al., 2022; Ouyang and Zhang, 2022; Song et al., 2022; Wang, 2022; Zhang et al., 2022; Zhao, 2022; Yan et al., 2023) comprising 4,473 participants reported the total efficiency rate, and the pooled data showed that XST improved the total efficiency rate (RR = 1.19; 95% CI [1.15 to 1.23]; *p* < 0.00001) (Supplementary Figure S9). Considering that high heterogeneity (I^2^ = 52%, *p* < 0.0001) could not be changed significantly through the sensitivity analysis (Supplementary Figure S10), we performed subgroup analyses, respectively, by the duration of treatment (7 days, RR = 1.17; 95% CI [1.02 to 1.34]; *p* = 0.03; 14 days, RR = 1.16; 95% CI [1.12 to 1.21]; *p* < 0.00001; 28 days, RR = 1.27; 95% CI [1.20 to 1.34]; *p* < 0.00001) (Supplementary Figure S11), by the dosage form (XST injection, RR = 1.18; 95% CI [1.14 to 1.22]; *p* < 0.00001; XST oral preparation, RR = 1.55; 95% CI [1.24 to 1.94]; *p* < 0.00001) (Supplementary Figure S12), by the combination treatment (conventional treatment, RR = 1.18; 95% CI [1.13 to 1.24]; *p* < 0.00001; neuroprotective agents plus conventional treatment, RR = 1.19; 95% CI [1.14 to 1.25]; *p* < 0.00001) (Supplementary Figure S13), and by the time of administration (treatment initiated within 72 h, RR = 1.19; 95% CI [1.15 to 1.23]; *p* < 0.00001; treatment initiated within 14 days [except for studies initiated within 72 h only], RR = 1.14; 95% CI [1.05 to 1.24]; *p* < 0.00001) (Supplementary Figure S14).

#### 3.5.5 Blood rheology indicators

The meta-analysis results of XST on HBV, LBV, FIB, PV, and Hct are shown in Supplementary Table S1 and Supplementary Figure S15. The detailed contents are presented in Supplementary Material.

#### 3.5.6 Adverse events

**FIGURE 7:**
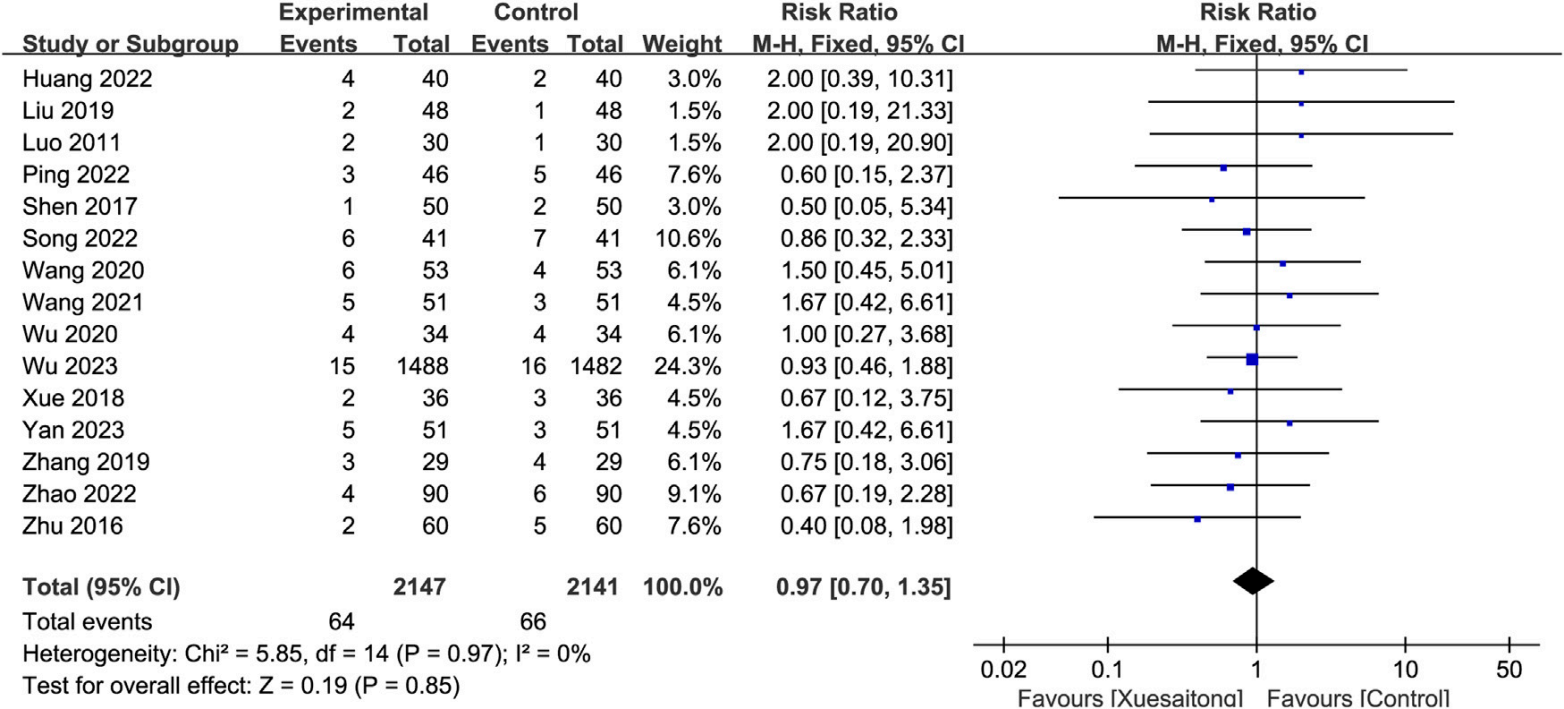
Forest plot for the effect of Xuesaitong on adverse events.

Of all studies, 15 studies (Luo et al., 2011; Zhu et al., 2016; Shen and Zhang, 2017; Xue, 2018; Liu et al., 2019; Zhang, 2019; Wang et al., 2020; Wu, 2020; Wang et al., 2021; Huang et al., 2022; Ping et al., 2022; Song et al., 2022; Zhao, 2022; Wu et al., 2023; Yan et al., 2023) comprising 4,288 cases reported adverse events. No heterogeneity was found (I^2^ = 0%, *p* = 0.97); thus, a fixed-effects model was adopted. There was no significant difference between the XST group and the control group (RR = 0.97; 95% CI [0.70 to 1.35]; *p* = 0.85) (Figure 7). No increased rate of adverse events was observed in patients who received XST treatment. Among the 16 studies, a total of 135 participants reported detailed information on adverse events before the end of the follow-up. Nausea, dizziness, and skin irritation were the most frequently reported adverse events.

### 3.6 Additional data from the latest large-scale RCT

Our review demonstrated that XST might have clinical efficacy in the improvement of activities of daily living and neurological impairment. Additional data from the latest large-scale RCT (Wu et al., 2023) reported the NIHSS score change from baseline to 90 days [XST: −4 (IQR −5 to −3); placebo: −4 (IQR −5 to −3); *p* = 0.02] and the BI score change from baseline to 90 days [XST: 15 (IQR, 5–35); placebo: 15 (IQR, 5–30); *p* = 0.006]. The evidence was substantial, indicating that XST was more effective in enhancing neurologic deficits. Notably, this trial provided new evidence of the symptomatic intracranial hemorrhage (XST: 1/1,488 (0.1%); placebo: 0/1,482 (0); *p* = 0.32), indicating that XST may not increase the risk of bleeding.

### 3.7 Publication bias

The funnel plot (Supplementary Figure S16) and statistical test indicated that no obvious publication bias was found in included trials regarding the BI score (Egger’s test, *p* = 0.441), the NIHSS score (Begg’s test, *p* = 0.358), HBV (Egger’s test, *p* = 0.193), LBV (Egger’s test, *p* = 0.478), FIB (Egger’s test, *p* = 0.774), PV (Egger’s test, *p* = 0.460), Hct (Egger’s test, *p* = 0.179) levels, and adverse events (Egger’s test, *p* = 0.099). However, a publication bias risk was present for the total efficiency rate (Begg’s test, *p* = 0.000). The publication bias of the long-term functional outcomes, all-cause mortality, and ESS score could not be estimated for only one or two included trials.

### 3.8 Quality of evidence

The certainty of the evidence of XST on adverse events was rated as “moderate”; that on functional independence, all-cause mortality, the BI score, and the NIHSS score was “low”; and that on the mRS score, the ESS score, the total efficiency rate, and blood rheology indicators was “very low” (Table 2). We judged the quality of evidence as moderate to very low, mainly due to the high risk of bias and the serious inconsistency.

**TABLE 2:** GRADE evidence profiles.

Outcomes	No. of participants (No. of studies)	Certainty assessment							Absolute effect (95% CI)	Certainty
		Study design	Risk of bias	Inconsistency	Indirectness	Imprecision	Other considerations	Relative effect (95% CI)		
mRS score	192 (1)	High	Serious^b^	Serious^c^	Not serious	Not serious	None	-	MD 0.67 lower (0.92–0.42 lower)	⊕○○○ Very low
Functional independence^a^	2966 (1)	High	Not serious	Serious^c^	Not serious	Not serious	None	RR 1.08 (1.05–1.12)	-	⊕⊕○○ Low
All-cause mortality	3162 (2)	High	Serious^b^	Serious^c^	Not serious	Not serious	None	RR 0.43 (0.06–2.93)	-	⊕⊕○○ Low
BI	1372 (12)	High	Serious^b^	Serious^c^	Not serious	Not serious	None	-	MD 10.17 higher (7.28–13.06 higher)	⊕⊕○○ Low
NIHSS	3385 (29)	High	Serious^b^	Serious^c^	Not serious	Not serious	None	-	MD 3.39 lower (3.94–2.84 lower)	⊕⊕○○ Low
ESS	50 (1)	High	Serious^b^	Serious^c^	Not serious	Serious	None	-	MD 11.85 higher (2.07–21.63 higher)	⊕○○○ Very low
Total efficiency rate	4473 (40)	High	Serious^b^	Serious^c^	Not serious	Not serious	Publication bias strongly suspected	RR 1.19 (1.15–1.23)	-	⊕○○○ Very low
HBV	1144 (11)	High	Serious^b^	Serious^c^	Not serious	Not serious	None	-	MD 0.86 lower (1.07–0.64 lower)	⊕○○○ Very low
LBV	1144 (11)	High	Serious^b^	Serious^c^	Not serious	Not serious	None	-	MD 1.55 lower (1.91–1.18 lower)	⊕○○○ Very low
FIB	676 (7)	High	Serious^b^	Serious^c^	Not serious	Not serious	None	-	MD 0.72 lower (1.11–0.34 lower)	⊕○○○ Very low
PV	1176 (12)	High	Serious^b^	Serious^c^	Not serious	Not serious	None	-	MD 0.39 lower (0.54–0.24 lower)	⊕○○○ Very low
Hct	578 (6)	High	Serious^b^	Serious^c^	Not serious	Not serious	None	-	MD 5.12 lower (6.09–4.14 lower)	⊕○○○ Very low
Adverse effects	4288 (15)	High	Serious^b^	Not serious	Not serious	Not serious	None	RR 0.97 (0.70–1.35)	-	⊕⊕⊕○ Moderate

Abbreviations: RCT, randomized controlled trial; RR, relative risk; MD, mean difference; mRS, modified Rankin Scale; BI, Barthel Index; NIHSS, National Institute of Health Stroke Scale; ESS, European Stroke Scale; HBV, whole blood high-cut viscosity; LBV, whole blood low-cut viscosity; FIB, fibrinogen; PV, plasma viscosity; Hct, hematocrit.

a The proportion of patients with functional independence (mRS ≤ 2).

b Poor methodological quality.

c Serious inconsistency.

## 4 Discussion

### 4.1 Summary of the main results

This meta-analysis consisted of 46 RCTs on the efficacy and safety of XST for patients with AIS, including a total of 7,957 participants and two dosage forms. Regarding the long-term functional outcomes, most initial RCTs did not prespecify or report on long-term functional outcomes, and only two trials reported relevant outcomes that could not be synthesized. One of the RCTs reported the proportion of functional independence at 90 days [XST: 1,328/1,487 (89.3%); placebo: 1,218/1,479 (82.4%); OR, 1.95; *p* < 0.001], while the other low-evidence quality study reported the mean of the mRS score. Additionally, pharmacological studies have proven that XST can promote the polarization of microglia to an M2 phenotype, inhibit neuronal cell death via the downregulation of the STAT3 signaling pathway, reduce Nogo-A expression, and inhibit the ROCKII pathway, exerting long-term neuroprotective effects (Li et al., 2019; Zhou et al., 2021). Even though we could not synthesize the effect sizes of the two studies, we believe that XST is highly likely to have a superior therapeutic benefit in the long-term functional outcomes. For the mRS, the FDA accepted the dichotomous approach as the primary outcome measure for subsequent AIS trials since it was convenient for physicians and researchers and had the advantage of being translatable into a number needed to treat (Broderick et al., 2017). We suggest that researchers should conduct relevant RCTs with more rigorous and internationally recognized methodological designs for better evidence synthesis and clinical practice in the future.

As for secondary outcomes, this study did not indicate that XST could reduce all-cause mortality by pooling a few corresponding data, while low-certainty evidence of most studies revealed that XST enhanced the total efficiency rate. Compared with other outcome indices, authors of previous studies seemed to prefer to use the total efficiency rate instead of an objective outcome index such as all-cause mortality, and we hold a dialectical perspective. The total efficiency might provide an intuitional impression of the outcomes. However, standardized approaches are not generally accepted and validated for interpretation, and it is an inadequate strategy to evaluate a composite endpoint as if it were a single primary endpoint (McCoy, 2018). The pooling data might lead to error accumulation of the total efficiency rate, and we recommend that future studies should avoid such a subjective outcome index, as to date, little guidance exists on how to interpret the aggregated endpoints (Armstrong and Westerhout, 2017). Additionally, low-certainty evidence suggested that XST improved the BI score and reduced the NIHSS score. These estimates might be very imprecise, as high heterogeneity existed and did not decrease after the application of sensitivity analyses and subgroup analyses. Furthermore, the subgroup analyses showed that regardless of the type of XST dosage form used in the acute phase of ischemic stroke, XST might be an effective alternative therapy in the improvement of the activities of daily living and neurological impairment at different durations of treatment. Notably, the improvement in neurological impairment and activities of daily living seemed to be more obvious in the XST with conventional treatment group than XST with neuroprotective agents plus conventional treatment. In addition, we detected that treatment initiated within 72 h showed more effective results according to the subgroup analysis of the total efficiency rate.

The safety outcomes of XST in patients treated for AIS remained unknown according to the previous meta-analyses. Our meta-analytical evidence from RCTs revealed that there was no significant difference in safety outcomes. The XST group and the control group both showed good tolerability, and the reported adverse reactions might be relevant to the disease or other therapeutic procedures. A large-scale, population-based post-marketing study showed that the XST injection is well tolerated and has favorable safety, with a mean cumulative medication time of 7.53 ± 5.39 days (He et al., 2020). However, most of the included RCTs used XST injection with a duration of 14 days or even 28 days, while no increasing adverse events were found. Furthermore, only one study (Wu et al., 2023) reported bleeding events, which limited us to drawing the relevant conclusion. Indeed, we look forward to a more rigorous design and more transparent reporting so that we can clarify the application of different dosage forms and specify the dosage and duration. Additionally, the latest large-scale RCT showed XST did not increase the risk of safety events [XST: 15/1,488 (1.0%); placebo: 16/1,482 (1.1%); *p* = 0.85], and we expect more reliable trials of the safety of XST in the future to inform this field.

### 4.2 Comparison with previous studies

Compared with the two previous reviews regarding the effectiveness of XST, the present systematic review and meta-analysis included all dosage forms of XST and more recent RCTs, especially the latest large-scale RCT from our team (Wu et al., 2023). Previous low-quality trials might have overestimated the efficacy of XST. In addition, the previous systematic reviews were merely concerned with the total effective rate, the NIHSS score, the CSS score, and blood rheology indicators. However, we attempted to explore whether XST could improve long-term functional outcomes and reduce all-cause mortality, which are more objective and vital for patients with AIS. The comparisons of the studies mentioned previously are shown in Table 3. We made efforts to contact the authors and tried to obtain the generation of random sequences through e-mail and telephone. Ultimately, we excluded the articles in which “selection of participants” or “retrospective analysis” was mentioned in addition to “randomization” if the authors were unavailable to provide the generation of random sequences. We aimed to provide this field with a more comprehensive and specific evaluation of XST for patients with AIS.

**TABLE 3:** Comparisons of several studies.

	2015 meta-analysis	2022 meta-analysis	2023 RCT	2023 meta-analysis
Number of included RCTs	23	8	NA	47
Improvement in functional status	NA	NA	mRS: Xuesaitong: 1,328/1,487 (89.3%); placebo: 1,218/1,479 (82.4%); OR, 1.95; *p* < 0.001	mRS: MD = −0.67; 95% CI [−0.92 to −0.42]; *p* < 0.00001; RR = 1.08; 95% CI [1.05 to 1.12]; *p* < 0.00001
All-cause mortality	NA	NA	Xuesaitong: 1/1,488 (0.0%); placebo: 2/1,482 (0.0%); OR, 0.50; *p* = 0.57	RR = 0.43; 95% CI [0.06 to 2.93]; *p* = 0.39
Improvement in activities of daily living	NA	NA	△BI: Xuesaitong: 15 (IQR, 5–35); placebo: 15 (IQR, 5–30); *p* = 0.006	BI: MD = 10.17; 95% CI [7.28 to 13.06]; *p* < 0.00001
Improvement in neurological impairment	MD = −4.35, 95% CI [−6.61, −2.08], *p* = 0.0002	NIHSS: MD = −3.22, 95% CI [-4.52, −1.92], *p* < 0.00001CSS: MD = −6.53, 95% CI [−9.07, −3.99], *p* < 0.00001	△NIHSS: Xuesaitong: −4 (IQR -5 to −3); placebo: −4 (IQR -5 to −3); *p* = 0.02	NIHSS: MD = −3.39; 95% CI [−3.94 to −2.84]; *p* < 0.00001ESS: MD = 11.85; 95% CI [2.07 to 21.63]; *p* = 0.02
Total efficiency rate	RR = 1.21, 95% CI [1.16, 1.25], *p* < 0.00001	OR = 4.53, 95% CI [2.85,7.19], *p* < 0.0001	NA	RR = 1.19; 95% CI [1.15 to 1.23]; *p* < 0.00001
Blood rheology indicators	PV: MD = −0.14, 95% CI [−0.21, −0.08], *p* < 0.00001	HBV: MD = −0.63, 95% CI [-0.73, −0.53], *p* = 0.84LBV: MD = −0.37, 95% CI [-0.56, −0.19], *p* = 0.96FIB: MD = −23.78, 95% CI [-28.57, −18.99], *p* = 1.00PV: MD = −0.74, 95% CI [-0.96, −0.51], *p* < 0.00001Hct: MD = −2.76, 95% CI [-3.16, −2.36], *p* = 0.96	NA	HBV: MD = −0.86, 95% CI [-1.07, −0.64], *p* < 0.00001LBV: MD = −1.55, 95% CI [-1.91, −1.18], *p* = 0.0002FIB: MD = −0.72, 95% CI [-1.11, −0.34], *p* < 0.00001PV: MD = −0.39, 95% CI [-0.54, −0.24], *p* < 0.00001.Hct: MD = −5.12, 95% CI [-6.09, −4.14], *p* = 0.0001
Adverse effects	NA	NA	Xuesaitong: 15/1,488 (1.0%); placebo: 16/1,482 (1.1%); OR, 0.93; *p* = 0.85	RR = 0.97; 95% CI [0.70 to 1.35]; *p* = 0.85

RCT, randomized controlled trial; RR, relative risk; MD, mean difference; CI, confidence interval; mRS, modified Rankin Scale; BI, Barthel Index; NIHSS, National Institute of Health Stroke Scale; ESS, European Stroke Scale; HBV, whole blood high-cut viscosity; LBV, whole blood low-cut viscosity; FIB, fibrinogen; PV, plasma viscosity; Hct, hematocrit.

The latest systematic review and meta-analysis (Geng et al., 2022) published in 2022 synthetically assessed the efficacy and safety of XST oral preparation, including eight published RCTs up to August 2021. However, these eight studies were excluded during our screening for the following reasons: probably not RCTs (Li et al., 2013; Wang et al., 2017a) (n = 2), non-target population (Li and Liang, 2002; Mi and Wang, 2009) (n = 2), unclear onset time (Liu et al., 2005; Lu, 2010; Chang and He, 2017) (n = 3), and unavailable full-text report (Lin, 2007) (n = 1). Among the 23 RCTs included in the meta-analysis published in 2015 (Zhang et al., 2015), only one RCT (Fu et al., 2011) overlapped with the 46 studies included in our study. We excluded the other 22 RCTs for the following reasons: probably not RCTs (Yuan, 2003; Li and Qin, 2006; Yuan and Jiang, 2006; Wang and Li, 2007; Zhang and Zhang, 2008; Duan and Ai, 2009; Wang et al., 2011a; Yang and Cheng, 2012; Song, 2013) (n = 9), wrong randomization (Zhao, 2006; Rong and Zhi, 2008; He et al., 2011) (n = 3), inappropriate intervention (He, 2006; Wang, 2006; Li and Jiang, 2007; Wang, 2007; Zi et al., 2008; Ma and Wang, 2009) (n = 6), non-RCT (Zhang and Zhang, 2003) (n = 1), and unavailable full-text report (Li et al., 1999; Li, 2003; Cai, 2011) (n = 3). Although we attempted to contact the authors during our procedure, the information was still unavailable.

### 4.3 Limitations

Our study has some potential limitations. We pooled the data of the NIHSS score, the BI score, the total efficiency rate, and blood rheology indicators on conditions of significant statistical heterogeneity being observed, which lowered the evidence grade. This is likely because acceptable clinical heterogeneity existed in several aspects of the included studies, such as age, sex, onset time, cointerventions, treatment duration, and follow-up period. Although sensitivity and subgroup analyses were performed, confounding statistical results caused by heterogeneity could not be completely excluded. In addition, only two studies reported long-term functional outcomes that could not be synthesized, and we expect new relevant trials to update the meta-analysis. In addition, we expected to evaluate the XST administration during the acute phase of ischemic stroke (within 14 days of onset), but most of the included studies involved participants within 72 h of onset. We found the early time of XST administration might be associated with a higher total efficiency rate, and we failed to draw more conclusions due to the lack of relevant data. Furthermore, almost all of the included studies were at “high risk of bias,” which limited the interpretation of the previous results and further clinical application. We will be monitoring large-scale RCTs of XST to update this systematic review and meta-analysis if any high-quality trial emerges. Although we conducted this review rigorously and systematically, the results should be interpreted with caution before being recommended for clinical practice.

### 4.4 Implications for future research

Well-designed and properly conducted RCTs provide the gold standard for producing primary evidence, and fully reporting trial outcomes is vital for result-replication and knowledge-synthesis efforts (Butcher et al., 2022). Poorly reported findings have affected the conclusions drawn from systematic reviews and meta-analyses (Mayo-Wilson et al., 2017). We suggest that future RCTs register protocols prospectively and report the prespecified outcomes rigorously according to the CONSORT-CHM Formulas 2017 (Cheng et al., 2017). Similar to this review, future studies should strictly apply and transparently report the allocation concealment mechanism and double-blind methods. In addition, researchers should take into consideration the most appropriate and scientific method of aggregation of the outcomes, devoting attention to subsequent evidence synthesis and informing evidence-based clinical decision-making. If researchers have to use the composite outcome, it is advisable to determine an acknowledged definition of the composite outcome and all individual components of the composite outcome. Furthermore, high-quality evidence of the effectiveness of XST in patients with AIS is still insufficient, and the efficacy and safety of XST for AIS with proper intervention and long-term follow-up should be investigated to provide more robust and objective evidence.

## 5 Conclusion

In conclusion, the present systematic review and meta-analysis of 46 RCTs reveals that the administration of XST within 14 days for AIS is associated with favorable long-term functional outcomes. Additionally, XST can improve activities of daily living, alleviate neurological deficits, and has good tolerability. Nevertheless, the current evidence is too weak and needs to be proven by further high-quality evidence. The positive effects have been restricted by the poor methodological quality and the high risk of bias, weakening the confidence in evidence synthesis. Considering that the current evidence is too weak and that XST is a promising agent against AIS, researchers should conduct RCTs with more rigorous methodological designs and more transparent reporting to provide more evidence with moderate to high certainty.

## Data Availability

The original contributions presented in the study are included in the article/Supplementary Material; further inquiries can be directed to the corresponding authors.

## Abbreviations

AIS, acute ischemic stroke; BI, Barthel Index; CI, confidence interval; CNKI, China National Knowledge Infrastructure; CNS, Canadian Neurological Scale; ESS, European Stroke Scale; FIB, fibrinogen; GOS, Glasgow Outcome Scale; HBV, whole blood high-cut viscosity; Hct, hematocrit; LBV, whole blood low-cut viscosity; MD, mean difference; MESSS, Modified Edinburgh–Scandinavian Stroke Scale; mRS, modified Rankin Scale; NIHSS, National Institute of Health Stroke Scale; PNSs, *Panax notoginseng* saponins; PV, plasma viscosity; RCTs, randomized controlled trials; RR, relative risk; SSS, Scandinavian Stroke Scale; VIP, Chinese Science and Technology Journals Database; and XST, Xuesaitong.
