# Supplementary material for: Efficacy and safety of Panax notoginseng saponins (Xuesaitong) for patients with acute ischemic stroke: a systematic review and meta-analysis of randomized controlled trials
Source: Front Pharmacol. 2023 Oct 16;14:1280559. doi: 10.3389/fphar.2023.1280559 (PMC10614024; doi:10.3389/fphar.2023.1280559)

Supplementary Material

Title: Efficacy and safety of Panax notoginseng Saponins (Xuesaitong) for patients with acute ischemic stroke: A systematic review and meta-analysis of randomized controlled trials

- **Section I**

Preferred Reporting Items for Systematic Reviews and Meta-Analyses (PRISMA) 2020 checklist.

- **Section II**

Searching strategies

- **Section III**

Blood rheology indicators
Supplementary Table 1 Meta-analysis results of HBV, LBV, FIB, PV and Hct in two groups of acute ischemic stroke patients.

- **Section IV**

Supplementary Figure 1. The risk of bias summary for each study.
Supplementary Figure 2. Forest plot of subgroup analysis by duration of treatment for the effect of Xuesaitong on Barthel Index score.
Supplementary Figure 3. Forest plot of subgroup analysis by the combination treatment for the effect of Xuesaitong on Barthel Index score.
Supplementary Figure 4. Forest plot of sensitivity analysis for the effect of Xuesaitong on National Institute of Health Stroke Scale score.
Supplementary Figure 5. Forest plot of subgroup analysis by duration of treatment for the effect of Xuesaitong on National Institute of Health Stroke Scale score.
Supplementary Figure 6. Forest plot of subgroup analysis by dosage form for the effect of Xuesaitong on National Institute of Health Stroke Scale score.
Supplementary Figure 7. Forest plot of subgroup analysis by the combination treatment for the effect of Xuesaitong on National Institute of Health Stroke Scale score.
Supplementary Figure 8. Forest plot for the effect of Xuesaitong on European Stroke Scale score.
Supplementary Figure 9. Forest plot for the effect of Xuesaitong on total efficiency rate.
Supplementary Figure 10. Forest plot sensitivity analysis for the effect of Xuesaitong on total efficiency rate.

Supplementary Figure 11. Forest plot of subgroup analysis by duration of treatment for the effect of Xuesaitong on total efficiency rate.
Supplementary Figure 12. Forest plot of subgroup analysis by dosage form for the effect of Xuesaitong on total efficiency rate.
Supplementary Figure 13. Forest plot of subgroup analysis by the combination treatment for the effect of Xuesaitong on total efficiency rate.
Supplementary Figure 14. Forest plot of subgroup analysis by the time of administration for the effect of Xuesaitong on total efficiency rate.
Supplementary Figure 15. Forest plot for the effect of Xuesaitong on (A) Whole blood high-cut viscosity, (B) Whole blood low-cut viscosity, (C) Fibrinogen, (D) Plasma viscosity, (E) Hematocrit.
Supplementary Figure 16. Funnel plots of (A) Barthel Index score, (B) National Institute of Health Stroke Scale score, (C) Total efficiency rate, (D) Whole blood high-cut viscosity, (E) Whole blood low-cut viscosity, (F) Plasma viscosity, (G) Adverse events.

Section I. PRISMA 2020 Checklist

Section and Topic	Item #	Checklist item	Location where item is reported
**TITLE**			
Title	1	Identify the report as a systematic review.	Page 1, line 1
**ABSTRACT**			
Abstract	2	See the PRISMA 2020 for Abstracts checklist.	Page 1, line 24
**INTRODUCTION**			
Rationale	3	Describe the rationale for the review in the context of existing knowledge.	Page 3, line 106
Objectives	4	Provide an explicit statement of the objective(s) or question(s) the review addresses.	Page 3, line 103
**METHODS**			
Eligibility criteria	5	Specify the inclusion and exclusion criteria for the review and how studies were grouped for the syntheses.	Page 3, line 119
Information sources	6	Specify all databases, registers, websites, organisations, reference lists and other sources searched or consulted to identify studies. Specify the date when each source was last searched or consulted.	Page 3, line 110
Search strategy	7	Present the full search strategies for all databases, registers and websites, including any filters and limits used.	Supplementary Files
Selection process	8	Specify the methods used to decide whether a study met the inclusion criteria of the review, including how many reviewers screened each record and each report retrieved, whether they worked independently, and if applicable, details of automation tools used in the process.	Page 4, line 141
Data collection process	9	Specify the methods used to collect data from reports, including how many reviewers collected data from each report, whether they worked independently, any processes for obtaining or confirming data from study investigators, and if applicable, details of automation tools used in the process.	Page 4, line 146
Data items	10a	List and define all outcomes for which data were sought. Specify whether all results that were compatible with each outcome domain in each study were sought (e.g. for all measures, time points, analyses), and if not, the methods used to decide which results to collect.	Page 4, line 129
	10b	List and define all other variables for which data were sought (e.g. participant and intervention characteristics, funding sources). Describe any assumptions made about any missing or unclear information.	Page 3, line 148
Study risk of bias assessment	11	Specify the methods used to assess risk of bias in the included studies, including details of the tool(s) used, how many reviewers assessed each study and whether they worked independently, and if applicable, details of automation tools used in the process.	Page 4, line 152
Effect measures	12	Specify for each outcome the effect measure(s) (e.g. risk ratio, mean difference) used in the synthesis or presentation of results.	Page 5, line 159
Synthesis methods	13a	Describe the processes used to decide which studies were eligible for each synthesis (e.g. tabulating the study intervention characteristics and comparing against the planned groups for each synthesis (item #5)).	Page 5, line 159
	13b	Describe any methods required to prepare the data for presentation or synthesis, such as handling of missing summary statistics, or data conversions.	N/A
	13c	Describe any methods used to tabulate or visually display results of individual studies and syntheses.	Page 5, line 159
	13d	Describe any methods used to synthesize results and provide a rationale for the choice(s). If meta-analysis was performed, describe the model(s), method(s) to identify the presence and extent of statistical heterogeneity, and software package(s) used.	Page 5, line 164

Section and Topic	Item #	Checklist item	Location where item is reported
	13e	Describe any methods used to explore possible causes of heterogeneity among study results (e.g. subgroup analysis, meta-regression).	Page 5, line 168
	13f	Describe any sensitivity analyses conducted to assess robustness of the synthesized results.	Page 5, line 168
Reporting bias assessment	14	Describe any methods used to assess risk of bias due to missing results in a synthesis (arising from reporting biases).	N/A
Certainty assessment	15	Describe any methods used to assess certainty (or confidence) in the body of evidence for an outcome.	N/A
**RESULTS**			
Study selection	16a	Describe the results of the search and selection process, from the number of records identified in the search to the number of studies included in the review, ideally using a flow diagram.	Page 5, line 186
	16b	Cite studies that might appear to meet the inclusion criteria, but which were excluded, and explain why they were excluded.	N/A
Study characteristics	17	Cite each included study and present its characteristics.	Page 23, line 810
Risk of bias in studies	18	Present assessments of risk of bias for each included study.	Page 6, line 200
Results of individual studies	19	For all outcomes, present, for each study: (a) summary statistics for each group (where appropriate) and (b) an effect estimate and its precision (e.g. confidence/credible interval), ideally using structured tables or plots.	Page 29, line 824
Results of syntheses	20a	For each synthesis, briefly summarise the characteristics and risk of bias among contributing studies.	Page 6, line 219
	20b	Present results of all statistical syntheses conducted. If meta-analysis was done, present for each the summary estimate and its precision (e.g. confidence/credible interval) and measures of statistical heterogeneity. If comparing groups, describe the direction of the effect.	Page 6, line 219
	20c	Present results of all investigations of possible causes of heterogeneity among study results.	Page 10, line 409
	20d	Present results of all sensitivity analyses conducted to assess the robustness of the synthesized results.	N/A
Reporting biases	21	Present assessments of risk of bias due to missing results (arising from reporting biases) for each synthesis assessed.	N/A
Certainty of evidence	22	Present assessments of certainty (or confidence) in the body of evidence for each outcome assessed.	N/A
**DISCUSSION**			
Discussion	23a	Provide a general interpretation of the results in the context of other evidence.	Page 9, line 324
	23b	Discuss any limitations of the evidence included in the review.	Page 10, line 404
	23c	Discuss any limitations of the review processes used.	Page 10, line 404
	23d	Discuss implications of the results for practice, policy, and future research.	Page 11, line 421
**OTHER INFORMATION**			
Registration and protocol	24a	Provide registration information for the review, including register name and registration number, or state that the review was not registered.	Page 3, line 109
	24b	Indicate where the review protocol can be accessed, or state that a protocol was not prepared.	N/A
	24c	Describe and explain any amendments to information provided at registration or in the protocol.	N/A

Section and Topic	Item #	Checklist item	Location where item is reported
Support	25	Describe sources of financial or non-financial support for the review, and the role of the funders or sponsors in the review.	Page 12, line 458
Competing interests	26	Declare any competing interests of review authors.	Page 11, line 449
Availability of data, code and other materials	27	Report which of the following are publicly available and where they can be found: template data collection forms; data extracted from included studies; data used for all analyses; analytic code; any other materials used in the review.	N/A

Section II. Searching strategies

Searching Strategies (PubMed)

Search number	Query
1	("Ischemic Stroke"[Mesh]) OR (Ischemic Strokes) OR (Stroke, Ischemic) OR (Ischaemic Stroke) OR (Ischaemic Strokes) OR (Stroke, Ischaemic) OR (Cryptogenic Ischemic Stroke) OR (Cryptogenic Ischemic Strokes) OR (Ischemic Stroke, Cryptogenic) OR (Stroke, Cryptogenic Ischemic) OR (Cryptogenic Stroke) OR (Cryptogenic Strokes) OR (Stroke, Cryptogenic) OR (Cryptogenic Embolism Stroke) OR (Cryptogenic Embolism Strokes) OR (Embolism Stroke, Cryptogenic) OR (Stroke, Cryptogenic Embolism) OR (Wake-up Stroke) OR (Stroke, Wake-up) OR (Wake up Stroke) OR (Wake-up Strokes) OR (Acute Ischemic Stroke) OR (Acute Ischemic Strokes) OR (Ischemic Stroke, Acute) OR (Stroke, Acute Ischemic)
2	(Xuesaitong[Title/Abstract]) OR (Xue-Sai-Tong[Title/Abstract]) OR (Panax notoginseng saponins[Title/Abstract])
3	(randomized controlled trial[Title/Abstract]) OR (randomized[Title/Abstract]) OR (placebo[Title/Abstract])
4	#1 AND #2 AND #3

Searching Strategies (Embase)

Search number	Query
**1**	'ischemic stroke':ab,ti OR 'ischemic strokes':ab,ti OR 'stroke, ischemic':ab,ti OR 'ischaemic stroke':ab,ti OR 'ischaemic strokes':ab,ti OR 'stroke, ischaemic':ab,ti OR 'cryptogenic ischemic stroke':ab,ti OR 'cryptogenic ischemic strokes':ab,ti OR 'ischemic stroke, cryptogenic':ab,ti OR 'stroke, cryptogenic ischemic':ab,ti OR 'cryptogenic stroke':ab,ti OR 'cryptogenic strokes':ab,ti OR 'stroke, cryptogenic':ab,ti OR 'cryptogenic embolism stroke':ab,ti OR 'cryptogenic embolism strokes':ab,ti OR 'embolism stroke, cryptogenic':ab,ti OR 'stroke, cryptogenic embolism':ab,ti OR 'wake-up stroke':ab,ti OR 'stroke, wake-up':ab,ti OR 'wake up stroke':ab,ti OR 'wake-up strokes':ab,ti OR 'acute ischemic stroke':ab,ti OR 'acute ischemic strokes':ab,ti OR 'ischemic stroke, acute':ab,ti OR 'stroke, acute ischemic':ab,ti
**2**	'Xuesaitong':ab,ti OR 'Xue-Sai-Tong':ab,ti OR 'Panax notoginseng saponins':ab,ti
**3**	'randomized controlled trial':ab,ti OR 'randomized':ab,ti OR 'placebo':ab,ti
**4**	#1 AND #2 AND #3

Searching Strategies (the Cochrane library)

Search number	Query
**1**	(Ischemic Stroke):ab,ti,kw OR (Ischemic Strokes):ab,ti,kw (Stroke, Ischemic):ab,ti,kw OR (Ischaemic Stroke):ab,ti,kw OR (Ischaemic Strokes):ab,ti,kw OR (Stroke, Ischaemic):ab,ti,kw OR (Cryptogenic Ischemic Stroke):ab,ti,kw OR (Cryptogenic Ischemic Strokes):ab,ti,kw OR (Ischemic Stroke, Cryptogenic):ab,ti,kw OR (Stroke, Cryptogenic Ischemic):ab,ti,kw OR (Cryptogenic Stroke):ab,ti,kw OR (Cryptogenic Strokes):ab,ti,kw OR (Stroke, Cryptogenic):ab,ti,kw OR (Cryptogenic Embolism Stroke):ab,ti,kw OR (Cryptogenic Embolism Strokes):ab,ti,kw OR (Embolism Stroke, Cryptogenic):ab,ti,kw OR (Stroke, Cryptogenic Embolism):ab,ti,kw OR (Wake-up Stroke):ab,ti,kw OR (Stroke, Wake-up):ab,ti,kw OR (Wake up Stroke):ab,ti,kw OR (Wake-up Strokes):ab,ti,kw OR (Acute Ischemic Stroke):ab,ti,kw OR (Acute Ischemic Strokes):ab,ti,kw OR (Ischemic Stroke, Acute):ab,ti,kw OR (Stroke, Acute Ischemic):ab,ti,kw
**2**	(Xuesaitong):ab,ti,kw OR (Xue-Sai-Tong):ab,ti,kw OR (Panax notoginseng saponins):ab,ti,kw
**3**	(randomized controlled trial):ab,ti,kw OR (randomized):ab,ti,kw OR (placebo):ab,ti,kw
**4**	#1 AND #2 AND #3

Searching Strategies (Web of Science)

Search number	Query
**1**	Ischemic Stroke (主题) or Ischemic Strokes (主题) or Stroke, Ischemic (主题) or Ischaemic Stroke (主题) or Ischaemic Strokes (主题) or Stroke, Ischaemic (主题) or Cryptogenic Ischemic Stroke (主题) or Cryptogenic Ischemic Strokes (主题) or Ischemic Stroke, Cryptogenic (主题) or Stroke, Cryptogenic Ischemic (主题) or Cryptogenic Stroke (主题) or Cryptogenic Strokes (主题) or Stroke, Cryptogenic (主题) or Cryptogenic Embolism Stroke (主题) or Cryptogenic Embolism Strokes (主题) or Embolism Stroke, Cryptogenic (主题) or Stroke, Cryptogenic Embolism (主题) or Wake-up Stroke (主题) or Stroke, Wake-up (主题) or Wake up Stroke (主题) or Wake-up Strokes (主题) or Acute Ischemic Stroke (主题) or Acute Ischemic Strokes (主题) or Ischemic Stroke, Acute (主题) or Stroke, Acute Ischemic (主题)
**2**	Xuesaitong (主题) or Xue-Sai-Tong (主题) or Panax notoginseng saponins (主题)
**3**	randomized controlled trial (主题) or randomized (主题) or placebo (主题)
**4**	#1 AND #2 AND #3

Searching Strategies (CNKI)

TKA =(‘血塞通’+‘络泰’) AND TKA=(‘脑梗死’+‘脑梗’+‘腔隙性脑梗’+ ‘腔梗’+‘脑栓塞’+‘脑血栓’+‘脑卒中’+‘卒中’+‘中风’+ ‘脑缺血’+‘脑血管病’+ ‘缺血性脑中风’ +‘缺血性脑卒中’+‘缺血性中风’+‘缺血性卒中’) AND TKA=（‘随机对照试验’+ ‘随机对照’+‘随机’+‘对照’+‘随机分配’+‘随机分组’+‘RCT’+‘安慰剂’）

Searching Strategies (WanFang)

(题名或关键词:(“血塞通” or “络泰”) and 题名或关键词: (“脑梗死” or “脑梗” or “腔隙性脑梗” or “腔梗” or “脑栓塞” or “脑血栓” or “脑卒中” or “卒中” or “中风” or “脑缺血” or “脑血管病” or “缺血性脑中风” or “缺血性脑卒中” or “缺血性中风” or “缺血性卒中”) and 题名或关键词: (“随机对照试验” or “随机对照” or “随机” or “对照” or “随机分配” or “随机分组” or “安慰剂” or “RCT”)) or(摘要:(“血塞通” or “络泰”) and 摘要:(“脑梗死” or “脑梗” or “腔隙性脑梗” or “腔梗” or “脑栓塞” or “脑血栓” or “脑卒中” or “卒中” or “中风” or “脑缺血” or “脑血管病” or “缺血性脑中风” or “缺血性脑卒中” or “缺血性中风”or “缺血性卒中”) and 摘要:(“随机对照试验” or “随机对照” or “随机” or “对照” or “随机分配” or “随机分组” or “安慰剂” or “RCT”))

Searching Strategies (VIP Database)

((M=(血塞通 OR 络泰)) OR R=(血塞通 OR 络泰)) AND ((M=(脑梗死 OR 脑梗 OR 腔隙性脑梗 OR 腔梗 OR 脑栓塞 OR 脑血栓 OR 脑卒中 OR 卒中 OR 中风 OR 脑缺血 OR 脑血管病 OR 缺血性脑中风 OR 缺血性脑卒中 OR 缺血性中风 OR 缺血性卒中)) OR R=(脑梗死 OR 脑梗 OR 腔隙性脑梗 OR 腔梗 OR 脑栓塞 OR 脑血栓 OR 脑卒中 OR 卒中 OR 中风 OR 脑缺血 OR 脑血管病 OR 缺血性脑中风 OR 缺血性脑卒中 OR 缺血性中风 OR 缺血性卒中)) AND ((M= (随机对照试验 OR 随机对照 OR 随机 OR 对照 OR 随机分配 OR 随机分组 OR 安慰剂 OR RCT)) OR R= (随机对照试验 OR 随机对照 OR 随机 OR 对照 OR 随机分配 OR 随机分组 OR 安慰剂 OR RCT))

Searching Strategies (SinoMed)

("血塞通"[常用字段:智能] OR "络泰"[常用字段:智能]) AND ("脑梗死"[常用字段:智能] OR "脑梗"[常用字段:智能] OR "腔隙性脑梗"[常用字段:智能] OR "腔梗"[常用字段:智能] OR "脑栓塞"[常用字段:智能] OR "脑血栓"[常用字段:智能] OR "脑卒中"[常用字段:智能] OR "卒中"[常用字段:智能] OR "中风"[常用字段:智能] OR "脑缺血"[常用字段:智能] OR "脑血管病"[常用字段:智能] OR "缺血性脑中风"[常用字段:智能] OR "缺血性脑卒中"[常用字段:智能] OR "缺血性中风"[常用字段:智能] OR "缺血性卒中"[常用字段:智能]) AND ("随机对照试验"[常用字段:智能] OR "随机对照"[常用字段:智能] OR "随机"[常用字段:智能] OR "对照"[常用字段:智能] OR "随机分配"[常用字段:智能] OR "随机分组"[常用字段:智能] OR "安慰剂"[常用字段:智能] OR "RCT"[常用字段:智能]

Section III.

Blood rheology indicators

The results showed that XST lowered blood rheology indicators, including HBV (MD = −0.86, 95% CI [-1.07, −0.64], p < 0.00001), LBV (MD = −1.55, 95% CI [-1.91, −1.18], p = 0.0002), FIB (MD = −0.72, 95% CI [-1.11, −0.34], p < 0.00001), PV (MD = −0.39, 95% CI [-0.54, −0.24], p < 0.00001), and Hct (MD = −5.12, 95% CI [-6.09, −4.14], p = 0.0001) (Fig. S15).

Supplementary Table 1 Meta-analysis results of HBV, LBV, FIB, PV and Hct in two groups of acute ischemic stroke patients.

Outcomes	Studies (n)	Cases (n)	Heterogeneity test		Effect model	MD (95% CI)	P value
			I^2	P value			
HBV	11	1144	82%	p < 0.00001	Random	-0.86 (-1.07, -0.64)	p < 0.00001
LBV	11	1144	73%	p < 0.00001	Random	-1.55 (-1.91, -1.18)	p < 0.00001
FIB	7	676	96%	p < 0.00001	Random	-0.72 (-1.11, -0.34)	p = 0.0002
PV	12	1176	98%	p < 0.00001	Random	-0.39 (-0.54, -0.24)	p < 0.00001
Hct	6	578	88%	p < 0.00001	Random	-5.12 (-6.09, -4.14)	p = 0.0001

Abbreviations: Whole blood high-cut viscosity (HBV), whole blood low-cut viscosity (LBV), fibrinogen (FIB), plasma viscosity (PV), hematocrit (Hct).

Section IV.

Supplementary Figure 2. The risk of bias summary for each study.

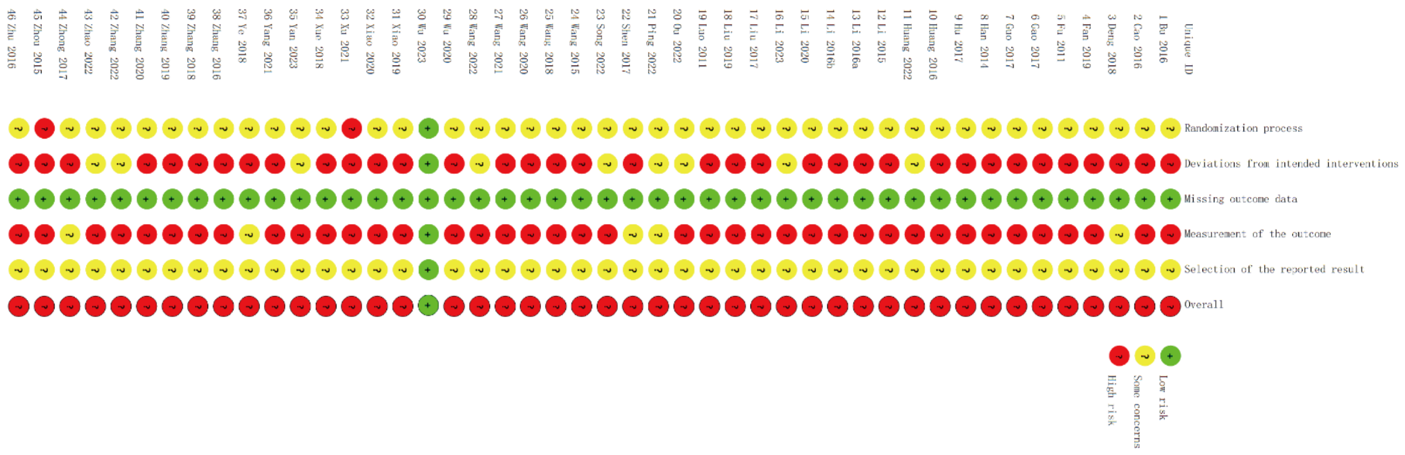

Supplementary Figure 2. Forest plot of subgroup analysis by duration of treatment for the effect of Xuesaitong on Barthel Index score.

Study or Subgroup	Experimental Mean	Experimental SD	Experimental Total	Control Mean	Control SD	Control Total	Weight	Mean Difference IV, Random, 95% CI
**1.3.1 14 days**								
Guo 2017	80	25	75	67	25	75	5.8%	13.00 [5.00, 21.00]
Liu 2019	80.93	5.48	48	73.89	5.29	48	9.9%	7.04 [4.89, 9.19]
Wang 2015	86.72	8.28	43	61.53	6.32	43	9.3%	25.19 [22.08, 28.30]
Wang 2018	85.32	1.34	43	74.33	12.37	43	8.9%	10.99 [7.27, 14.71]
Wang 2021	68.29	4.56	51	60.74	4.17	51	10.1%	7.55 [5.85, 9.25]
Xu 2021	92.44	4.32	90	87.15	3.68	90	10.3%	5.29 [4.12, 6.46]
Zhang 2018	80.65	24.48	46	63.55	24.75	46	4.6%	17.10 [7.04, 27.16]
Zhou 2015	79.6	24.83	65	62.36	24.83	65	5.5%	17.24 [8.70, 25.78]
**Subtotal (95% CI)**			**461**			**461**	**64.3%**	**12.40 [7.85, 16.95]**
Heterogeneity: Tau² = 36.16; Chi² = 150.29, df = 7 (P < 0.00001); I² = 95%								
Test for overall effect: Z = 5.34 (P < 0.00001)								
**1.3.2 28 days**								
Li 2016a	71.5	4.3	48	67.2	5.7	48	9.9%	4.30 [2.28, 6.32]
Wang 2022	60.89	2.21	39	50.46	2.34	39	10.3%	10.43 [9.42, 11.44]
Zhang 2022	74.98	18.69	96	67.49	20.16	96	7.6%	7.49 [1.99, 12.99]
Zhong 2017	66.15	12.45	42	62.42	11.15	42	7.9%	3.73 [-1.32, 8.78]
**Subtotal (95% CI)**			**225**			**225**	**35.7%**	**6.66 [2.42, 10.90]**
Heterogeneity: Tau² = 15.33; Chi² = 32.96, df = 3 (P < 0.00001); I² = 91%								
Test for overall effect: Z = 3.08 (P = 0.002)								
**Total (95% CI)**			**686**			**686**	**100.0%**	**10.17 [7.28, 13.06]**
Heterogeneity: Tau² = 20.88; Chi² = 186.08, df = 11 (P < 0.00001); I² = 94%								
Test for overall effect: Z = 6.89 (P < 0.00001)								
Test for subgroup differences: Chi² = 3.27, df = 1 (P = 0.07), I² = 69.4%								

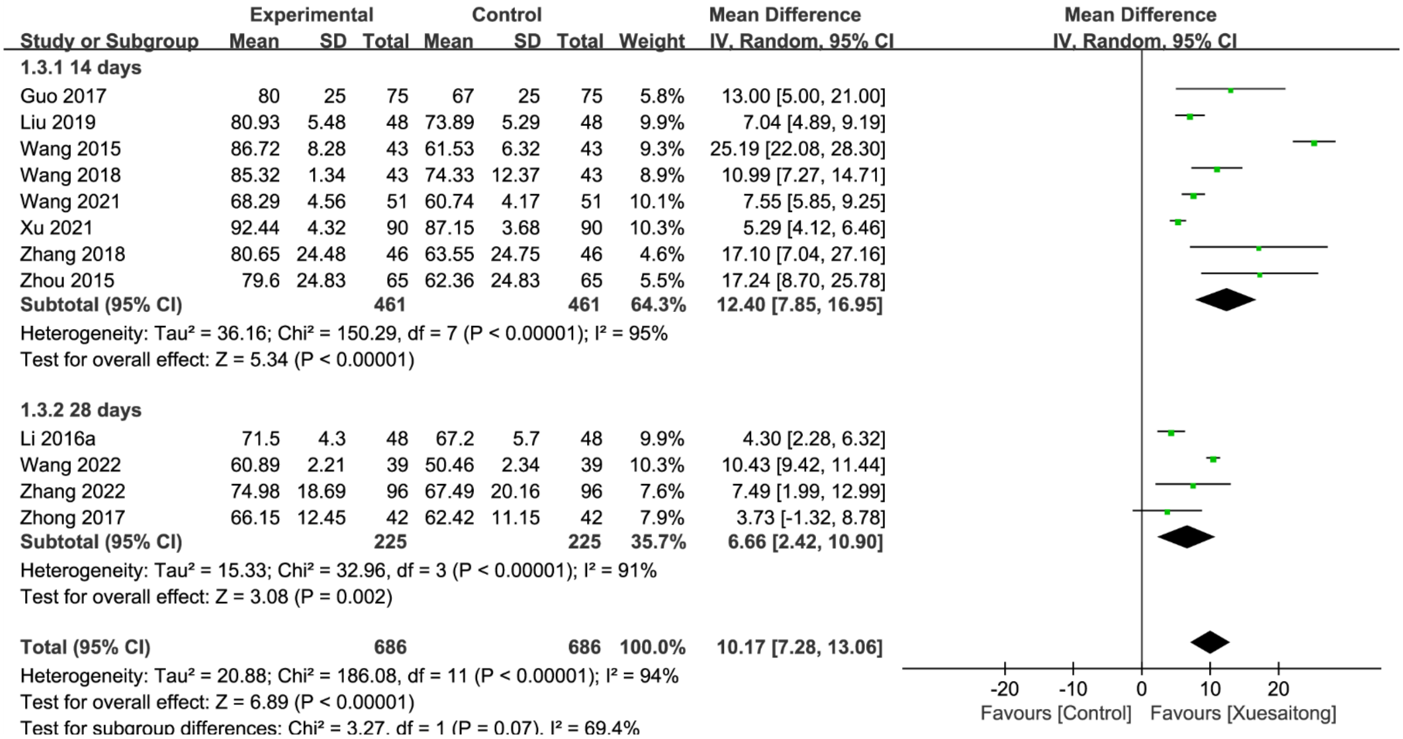

Supplementary Figure 3. Forest plot of subgroup analysis by the combination treatment for the effect of Xuesaitong on Barthel Index score.

	Experimental			Control				Mean Difference
Study or Subgroup	Mean	SD	Total	Mean	SD	Total	Weight	IV, Random, 95% CI
**1.30.1 Conventional treatment**								
Guo 2017	80	25	75	67	25	75	5.8%	13.00 [5.00, 21.00]
Wang 2015	86.72	8.28	43	61.53	6.32	43	9.3%	25.19 [22.08, 28.30]
Wang 2018	85.32	1.34	43	74.33	12.37	43	8.9%	10.99 [7.27, 14.71]
Wang 2021	68.29	4.56	51	60.74	4.17	51	10.1%	7.55 [5.85, 9.25]
Wang 2022	60.89	2.21	39	50.46	2.34	39	10.3%	10.43 [9.42, 11.44]
Xu 2021	92.44	4.32	90	87.15	3.68	90	10.3%	5.29 [4.12, 6.46]
Zhang 2018	80.65	24.48	46	63.55	24.75	46	4.6%	17.10 [7.04, 27.16]
Zhang 2022	74.98	18.69	96	67.49	20.16	96	7.6%	7.49 [1.99, 12.99]
Zhou 2015	79.6	24.83	65	62.36	24.83	65	5.5%	17.24 [8.70, 25.78]
**Subtotal (95% CI)**			**548**			**548**	**72.3%**	**12.17 [8.51, 15.84]**
Heterogeneity: Tau² = 24.99; Chi² = 161.19, df = 8 (P < 0.00001); I² = 95%								
Test for overall effect: Z = 6.51 (P < 0.00001)								
**1.30.2 Neuroprotective agents plus conventional treatment**								
Li 2016a	71.5	4.3	48	67.2	5.7	48	9.9%	4.30 [2.28, 6.32]
Liu 2019	80.93	5.48	48	73.89	5.29	48	9.9%	7.04 [4.89, 9.19]
Zhong 2017	66.15	12.45	42	62.42	11.15	42	7.9%	3.73 [-1.32, 8.78]
**Subtotal (95% CI)**			**138**			**138**	**27.7%**	**5.36 [3.23, 7.50]**
Heterogeneity: Tau² = 1.64; Chi² = 3.78, df = 2 (P = 0.15); I² = 47%								
Test for overall effect: Z = 4.92 (P < 0.00001)								
**Total (95% CI)**			**686**			**686**	**100.0%**	**10.17 [7.28, 13.06]**
Heterogeneity: Tau² = 20.88; Chi² = 186.08, df = 11 (P < 0.00001); I² = 94%								
Test for overall effect: Z = 6.89 (P < 0.00001)								
Test for subgroup differences: Chi² = 9.91, df = 1 (P = 0.002), I² = 89.9%								

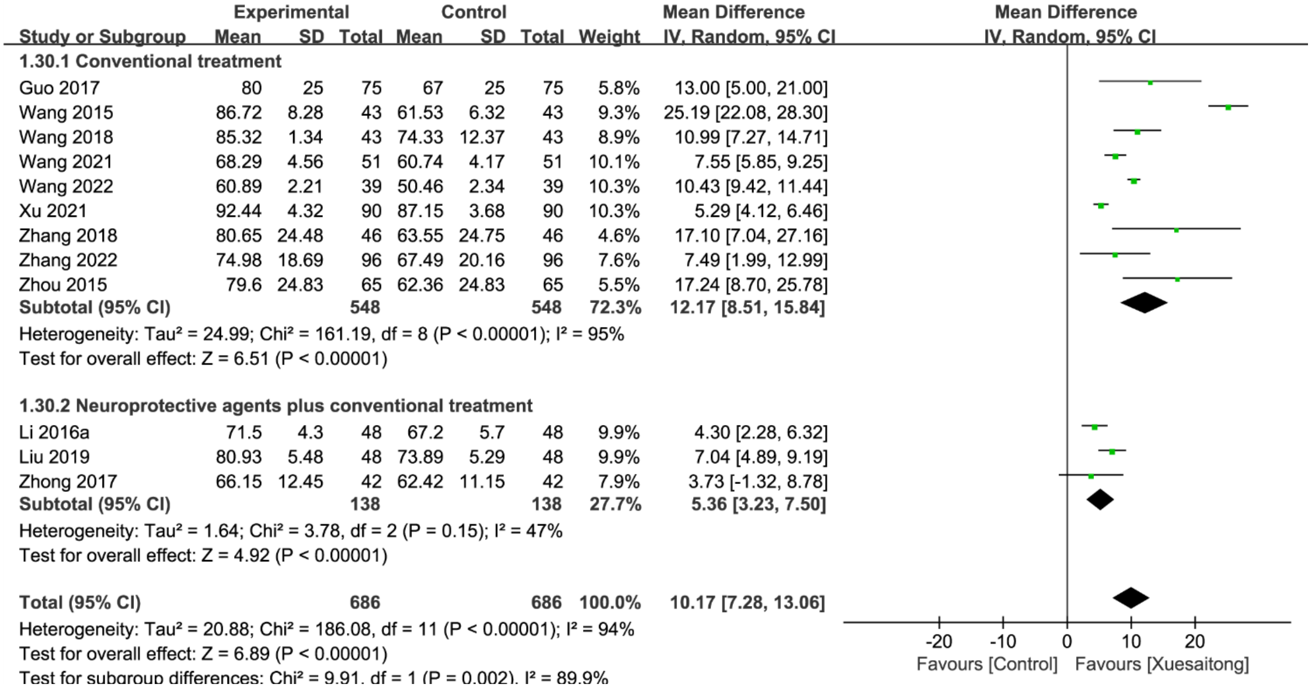

Supplementary Figure 4. Forest plot of sensitivity analysis for the effect of Xuesaitong on National Institute of Health Stroke Scale score.

	Experimental			Control				Mean Difference
Study or Subgroup	Mean	SD	Total	Mean	SD	Total	Weight	IV, Random, 95% CI
Cao 2016	5.54	0.37	40	7.43	0.71	40	4.3%	-1.89 [-2.14, -1.64]
Fan 2019	9.55	4.68	150	18.2	6.97	150	3.5%	-8.65 [-9.99, -7.31]
Guo 2017	11	4	75	16	5	75	3.3%	-5.00 [-6.45, -3.55]
Han 2014	11.48	3.47	41	18.29	4.26	40	3.1%	-6.81 [-8.50, -5.12]
Hu 2017	10.27	3.08	56	13.64	3.2	52	3.6%	-3.37 [-4.56, -2.18]
Huang 2022	6.35	2.27	40	7.08	2.25	40	3.8%	-0.73 [-1.72, 0.26]
Li 2015	8.32	1.54	62	10.22	1.45	62	4.2%	-1.90 [-2.43, -1.37]
Li 2016a	14.6	5.8	48	19.6	4.5	48	2.7%	-5.00 [-7.08, -2.92]
Li 2020	5.25	1.36	34	8.64	1.28	34	4.1%	-3.39 [-4.02, -2.76]
Liu 2017	14.13	4.2	120	17.75	5.23	120	3.6%	-3.62 [-4.82, -2.42]
Liu 2019	5.45	1.31	48	9.83	2.21	48	4.1%	-4.38 [-5.11, -3.65]
Luo 2011	5.02	3.3	30	7.03	2.89	30	3.2%	-2.01 [-3.58, -0.44]
Ping 2022	4.65	1.2	46	7.91	1.74	46	4.2%	-3.26 [-3.87, -2.65]
Wang 2015	2.78	5.79	43	6.06	6.74	43	2.1%	-3.28 [-5.94, -0.62]
Wang 2018	4.21	1.3	43	8.08	1.58	43	4.2%	-3.87 [-4.48, -3.26]
Wang 2020	13.57	3.16	53	16.39	3.27	53	3.6%	-2.82 [-4.04, -1.60]
Wang 2021	14.63	4.56	51	19.34	4.32	51	3.0%	-4.71 [-6.43, -2.99]
Wang 2022	4.33	0.55	39	8.11	0.56	39	4.3%	-3.78 [-4.03, -3.53]
Xu 2021	5.15	1.25	90	7.82	1.03	90	4.3%	-2.67 [-3.00, -2.34]
Xue 2018	10.16	2.12	36	15.25	3.22	36	3.5%	-5.09 [-6.35, -3.83]
Ye 2018	10.16	2.63	43	15.24	2.52	43	3.7%	-5.08 [-6.17, -3.99]
Zhang 2016	3.08	5.45	50	7.86	7.07	50	2.3%	-4.78 [-7.25, -2.31]
Zhang 2018	20.19	7.96	46	16.41	5.69	46	0.0%	3.78 [0.95, 6.61]
Zhang 2020	1.18	1.02	58	2.64	1.39	58	4.3%	-1.46 [-1.90, -1.02]
Zhang 2022	3.96	2.37	96	4.87	2.98	96	4.0%	-0.91 [-1.67, -0.15]
Zhao 2022	13.88	3.15	90	15.01	3.29	90	3.9%	-1.13 [-2.07, -0.19]
Zhong 2017	9.46	3.45	42	10.24	4.08	42	3.2%	-0.78 [-2.40, 0.84]
Zhou 2015	12.63	4.98	65	16.37	5.71	65	2.9%	-3.74 [-5.58, -1.90]
Zhu 2016	5.02	4.3	60	13.55	5.7	60	2.9%	-8.53 [-10.34, -6.72]
**Total (95% CI)**			**1649**			**1644**	**100.0%**	**-3.53 [-4.07, -2.99]**

Heterogeneity: Tau² = 1.75; Chi² = 440.13, df = 27 (P < 0.00001); I² = 94%
Test for overall effect: Z = 12.75 (P < 0.00001)

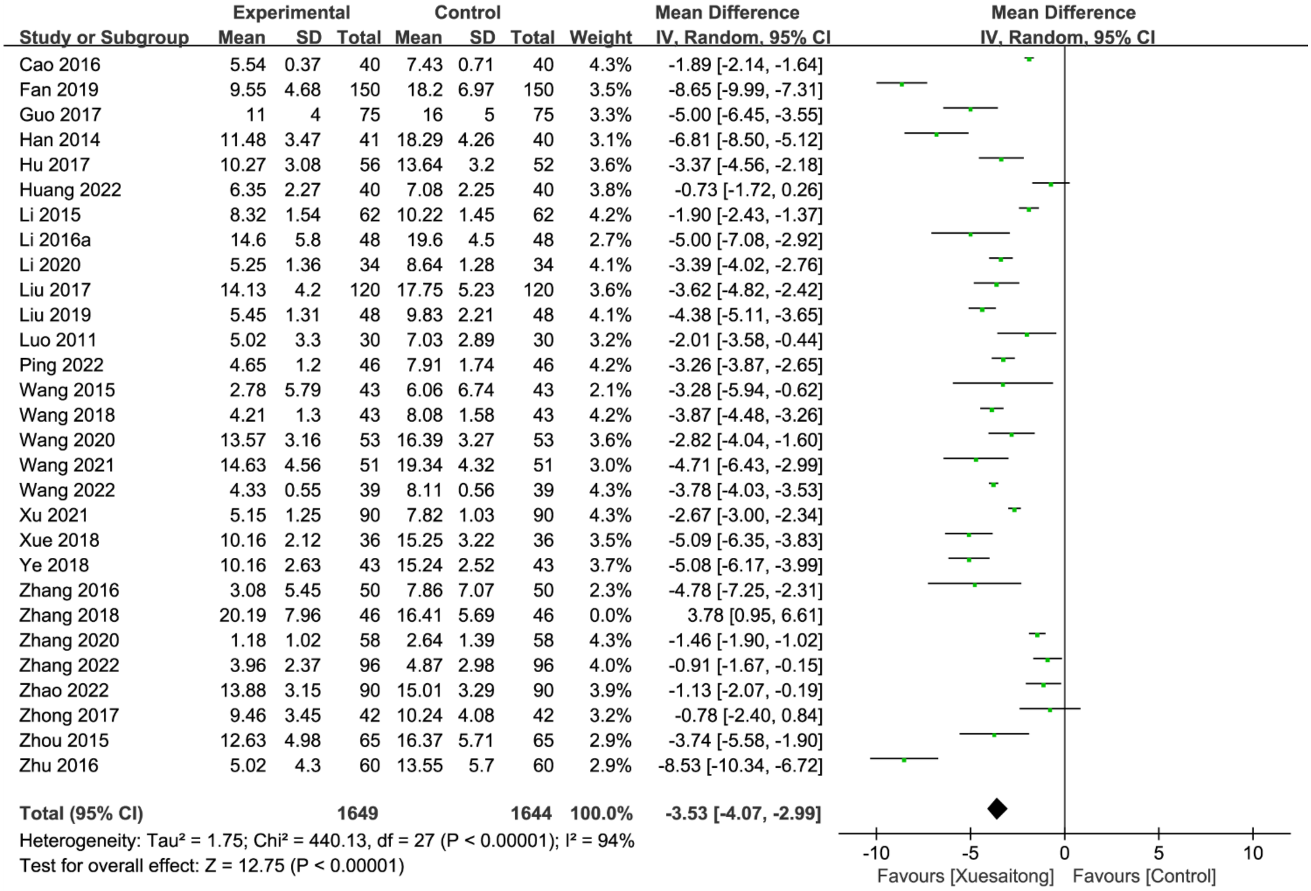

Supplementary Figure 5. Forest plot of subgroup analysis by duration of treatment for the effect of Xuesaitong on National Institute of Health Stroke Scale score.

Study or Subgroup	Experimental Mean	Experimental SD	Experimental Total	Control Mean	Control SD	Control Total	Weight	Mean Difference IV, Random, 95% CI
**1.4.1 14 days**								
Cao 2016	5.54	0.37	40	7.43	0.71	40	4.2%	-1.89 [-2.14, -1.64]
Fan 2019	9.55	4.68	150	18.2	6.97	150	3.4%	-8.65 [-9.99, -7.31]
Guo 2017	11	4	75	16	5	75	3.3%	-5.00 [-6.45, -3.55]
Han 2014	11.48	3.47	41	18.29	4.26	40	3.0%	-6.81 [-8.50, -5.12]
Huang 2022	6.35	2.27	40	7.08	2.25	40	3.7%	-0.73 [-1.72, 0.26]
Li 2015	8.32	1.54	62	10.22	1.45	62	4.1%	-1.90 [-2.43, -1.37]
Liu 2019	5.45	1.31	48	9.83	2.21	48	4.0%	-4.38 [-5.11, -3.65]
Luo 2011	5.02	3.3	30	7.03	2.89	30	3.2%	-2.01 [-3.58, -0.44]
Ping 2022	4.65	1.2	46	7.91	1.74	46	4.0%	-3.26 [-3.87, -2.65]
Wang 2015	2.78	5.79	43	6.06	6.74	43	2.1%	-3.28 [-5.94, -0.62]
Wang 2018	4.21	1.3	43	8.08	1.58	43	4.0%	-3.87 [-4.48, -3.26]
Wang 2020	13.57	3.16	53	16.39	3.27	53	3.5%	-2.82 [-4.04, -1.60]
Wang 2021	14.63	4.56	51	19.34	4.32	51	3.0%	-4.71 [-6.43, -2.99]
Xu 2021	5.15	1.25	90	7.82	1.03	90	4.2%	-2.67 [-3.00, -2.34]
Ye 2018	10.16	2.63	43	15.24	2.52	43	3.6%	-5.08 [-6.17, -3.99]
Zhang 2018	20.19	7.96	46	16.41	5.69	46	2.0%	3.78 [0.95, 6.61]
Zhang 2020	1.18	1.02	58	2.64	1.39	58	4.1%	-1.46 [-1.90, -1.02]
Zhao 2022	13.88	3.15	90	15.01	3.29	90	3.8%	-1.13 [-2.07, -0.19]
Zhou 2015	12.63	4.98	65	16.37	5.71	65	2.9%	-3.74 [-5.58, -1.90]
Zhu 2016	5.02	4.3	60	13.55	5.7	60	2.9%	-8.53 [-10.34, -6.72]
**Subtotal (95% CI)**			**1174**			**1173**	**69.2%**	**-3.42 [-4.12, -2.73]**
Heterogeneity: Tau² = 2.07; Chi² = 330.73, df = 19 (P < 0.00001); I² = 94%								
Test for overall effect: Z = 9.69 (P < 0.00001)								
**1.4.2 28 days**								
Hu 2017	10.27	3.08	56	13.64	3.2	52	3.6%	-3.37 [-4.56, -2.18]
Li 2016a	14.6	5.8	48	19.6	4.5	48	2.6%	-5.00 [-7.08, -2.92]
Li 2020	5.25	1.36	34	8.64	1.28	34	4.0%	-3.39 [-4.02, -2.76]
Liu 2017	14.13	4.2	120	17.75	5.23	120	3.5%	-3.62 [-4.82, -2.42]
Wang 2022	4.33	0.55	39	8.11	0.56	39	4.2%	-3.78 [-4.03, -3.53]
Xue 2018	10.16	2.12	36	15.25	3.22	36	3.5%	-5.09 [-6.35, -3.83]
Zhang 2016	3.08	5.45	50	7.86	7.07	50	2.3%	-4.78 [-7.25, -2.31]
Zhang 2022	3.96	2.37	96	4.87	2.98	96	3.9%	-0.91 [-1.67, -0.15]
Zhong 2017	9.46	3.45	42	10.24	4.08	42	3.1%	-0.78 [-2.40, 0.84]
**Subtotal (95% CI)**			**521**			**517**	**30.8%**	**-3.30 [-4.19, -2.42]**
Heterogeneity: Tau² = 1.39; Chi² = 69.60, df = 8 (P < 0.00001); I² = 89%								
Test for overall effect: Z = 7.35 (P < 0.00001)								
**Total (95% CI)**			**1695**			**1690**	**100.0%**	**-3.39 [-3.94, -2.84]**
Heterogeneity: Tau² = 1.83; Chi² = 461.25, df = 28 (P < 0.00001); I² = 94%								
Test for overall effect: Z = 12.13 (P < 0.00001)								
Test for subgroup differences: Chi² = 0.04, df = 1 (P = 0.83), I² = 0%								

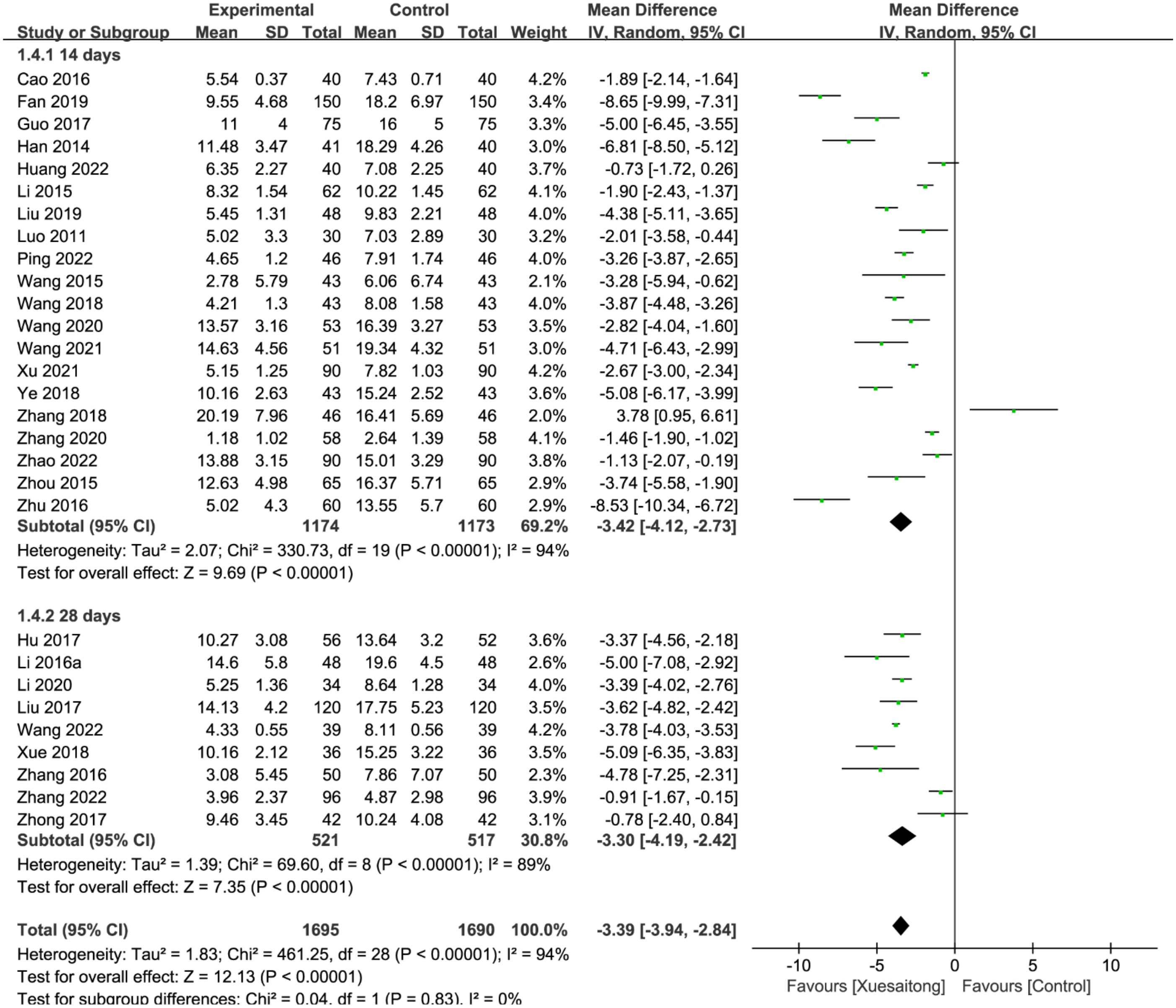

Supplementary Figure 6. Forest plot of subgroup analysis by dosage form for the effect of Xuesaitong on National Institute of Health Stroke Scale score.

	Experimental			Control				Mean Difference
Study or Subgroup	Mean	SD	Total	Mean	SD	Total	Weight	IV, Random, 95% CI
**1.5.1 Xuesaitong injection**								
Cao 2016	5.54	0.37	40	7.43	0.71	40	4.2%	-1.89 [-2.14, -1.64]
Fan 2019	9.55	4.68	150	18.2	6.97	150	3.4%	-8.65 [-9.99, -7.31]
Guo 2017	11	4	75	16	5	75	3.3%	-5.00 [-6.45, -3.55]
Han 2014	11.48	3.47	41	18.29	4.26	40	3.0%	-6.81 [-8.50, -5.12]
Hu 2017	10.27	3.08	56	13.64	3.2	52	3.6%	-3.37 [-4.56, -2.18]
Huang 2022	6.35	2.27	40	7.08	2.25	40	3.7%	-0.73 [-1.72, 0.26]
Li 2015	8.32	1.54	62	10.22	1.45	62	4.1%	-1.90 [-2.43, -1.37]
Li 2016a	14.6	5.8	48	19.6	4.5	48	2.6%	-5.00 [-7.08, -2.92]
Li 2020	5.25	1.36	34	8.64	1.28	34	4.0%	-3.39 [-4.02, -2.76]
Liu 2017	14.13	4.2	120	17.75	5.23	120	3.5%	-3.62 [-4.82, -2.42]
Liu 2019	5.45	1.31	48	9.83	2.21	48	4.0%	-4.38 [-5.11, -3.65]
Luo 2011	5.02	3.3	30	7.03	2.89	30	3.2%	-2.01 [-3.58, -0.44]
Ping 2022	4.65	1.2	46	7.91	1.74	46	4.0%	-3.26 [-3.87, -2.65]
Wang 2015	2.78	5.79	43	6.06	6.74	43	2.1%	-3.28 [-5.94, -0.62]
Wang 2018	4.21	1.3	43	8.08	1.58	43	4.0%	-3.87 [-4.48, -3.26]
Wang 2020	13.57	3.16	53	16.39	3.27	53	3.5%	-2.82 [-4.04, -1.60]
Wang 2021	14.63	4.56	51	19.34	4.32	51	3.0%	-4.71 [-6.43, -2.99]
Wang 2022	4.33	0.55	39	8.11	0.56	39	4.2%	-3.78 [-4.03, -3.53]
Xu 2021	5.15	1.25	90	7.82	1.03	90	4.2%	-2.67 [-3.00, -2.34]
Xue 2018	10.16	2.12	36	15.25	3.22	36	3.5%	-5.09 [-6.35, -3.83]
Ye 2018	10.16	2.63	43	15.24	2.52	43	3.6%	-5.08 [-6.17, -3.99]
Zhang 2018	20.19	7.96	46	16.41	5.69	46	2.0%	3.78 [0.95, 6.61]
Zhang 2020	1.18	1.02	58	2.64	1.39	58	4.1%	-1.46 [-1.90, -1.02]
Zhang 2022	3.96	2.37	96	4.87	2.98	96	3.9%	-0.91 [-1.67, -0.15]
Zhao 2022	13.88	3.15	90	15.01	3.29	90	3.8%	-1.13 [-2.07, -0.19]
Zhong 2017	9.46	3.45	42	10.24	4.08	42	3.1%	-0.78 [-2.40, 0.84]
Zhou 2015	12.63	4.98	65	16.37	5.71	65	2.9%	-3.74 [-5.58, -1.90]
Zhu 2016	5.02	4.3	60	13.55	5.7	60	2.9%	-8.53 [-10.34, -6.72]
**Subtotal (95% CI)**			**1645**			**1640**	**97.7%**	**-3.36 [-3.91, -2.80]**
Heterogeneity: Tau² = 1.83; Chi² = 458.89, df = 27 (P < 0.00001); I² = 94%								
Test for overall effect: Z = 11.88 (P < 0.00001)								
**1.5.2 Xuesaitong oral preparation**								
Zhang 2016	3.08	5.45	50	7.86	7.07	50	2.3%	-4.78 [-7.25, -2.31]
**Subtotal (95% CI)**			**50**			**50**	**2.3%**	**-4.78 [-7.25, -2.31]**
Heterogeneity: Not applicable								
Test for overall effect: Z = 3.79 (P = 0.0002)								
**Total (95% CI)**			**1695**			**1690**	**100.0%**	**-3.39 [-3.94, -2.84]**
Heterogeneity: Tau² = 1.83; Chi² = 461.25, df = 28 (P < 0.00001); I² = 94%								
Test for overall effect: Z = 12.13 (P < 0.00001)								
Test for subgroup differences: Chi² = 1.21, df = 1 (P = 0.27), I² = 17.4%								

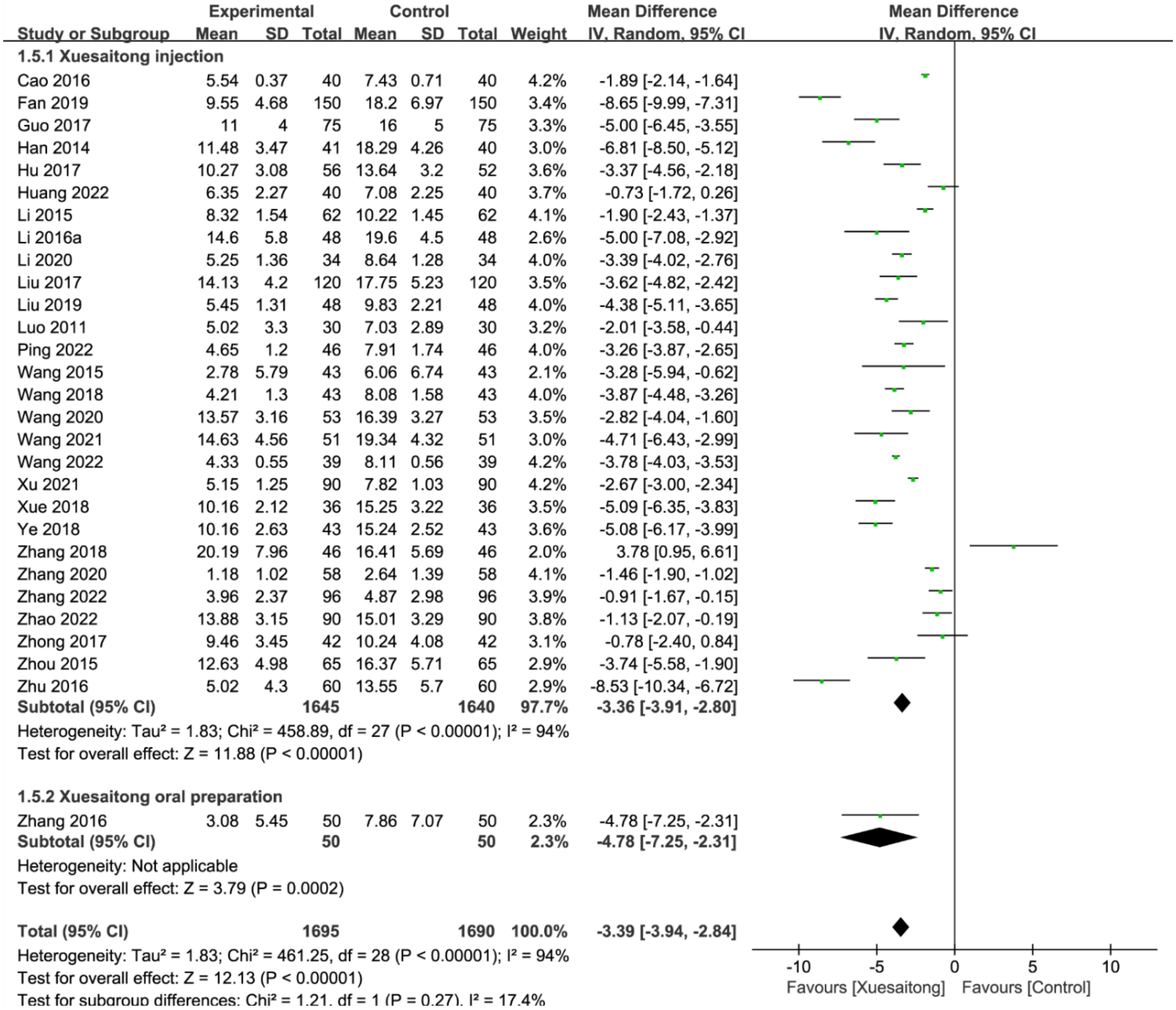

Supplementary Figure 7. Forest plot of subgroup analysis by the combination treatment for the effect of Xuesaitong on National Institute of Health Stroke Scale score.

	Experimental			Control				Mean Difference
Study or Subgroup	Mean	SD	Total	Mean	SD	Total	Weight	IV, Random, 95% CI
**1.29.1 Conventional treatment**								
Fan 2019	9.55	4.68	150	18.2	6.97	150	3.4%	-8.65 [-9.99, -7.31]
Guo 2017	11	4	75	16	5	75	3.3%	-5.00 [-6.45, -3.55]
Han 2014	11.48	3.47	41	18.29	4.26	40	3.0%	-6.81 [-8.50, -5.12]
Li 2015	8.32	1.54	62	10.22	1.45	62	4.1%	-1.90 [-2.43, -1.37]
Li 2020	5.25	1.36	34	8.64	1.28	34	4.0%	-3.39 [-4.02, -2.76]
Luo 2011	5.02	3.3	30	7.03	2.89	30	3.2%	-2.01 [-3.58, -0.44]
Wang 2015	2.78	5.79	43	6.06	6.74	43	2.1%	-3.28 [-5.94, -0.62]
Wang 2018	4.21	1.3	43	8.08	1.58	43	4.0%	-3.87 [-4.48, -3.26]
Wang 2021	14.63	4.56	51	19.34	4.32	51	3.0%	-4.71 [-6.43, -2.99]
Wang 2022	4.33	0.55	39	8.11	0.56	39	4.2%	-3.78 [-4.03, -3.53]
Xu 2021	5.15	1.25	90	7.82	1.03	90	4.2%	-2.67 [-3.00, -2.34]
Xue 2018	10.16	2.12	36	15.25	3.22	36	3.5%	-5.09 [-6.35, -3.83]
Ye 2018	10.16	2.63	43	15.24	2.52	43	3.6%	-5.08 [-6.17, -3.99]
Zhang 2016	3.08	5.45	50	7.86	7.07	50	2.3%	-4.78 [-7.25, -2.31]
Zhang 2018	20.19	7.96	46	16.41	5.69	46	2.0%	3.78 [0.95, 6.61]
Zhang 2020	1.18	1.02	58	2.64	1.39	58	4.1%	-1.46 [-1.90, -1.02]
Zhang 2022	3.96	2.37	96	4.87	2.98	96	3.9%	-0.91 [-1.67, -0.15]
Zhou 2015	12.63	4.98	65	16.37	5.71	65	2.9%	-3.74 [-5.58, -1.90]
**Subtotal (95% CI)**			**1052**			**1051**	**61.0%**	**-3.58 [-4.30, -2.86]**
Heterogeneity: Tau² = 1.95; Chi² = 289.56, df = 17 (P < 0.00001); I² = 94%								
Test for overall effect: Z = 9.74 (P < 0.00001)								
**1.29.2 Neuroprotective agents plus conventional treatment**								
Cao 2016	5.54	0.37	40	7.43	0.71	40	4.2%	-1.89 [-2.14, -1.64]
Hu 2017	10.27	3.08	56	13.64	3.2	52	3.6%	-3.37 [-4.56, -2.18]
Huang 2022	6.35	2.27	40	7.08	2.25	40	3.7%	-0.73 [-1.72, 0.26]
Li 2016a	14.6	5.8	48	19.6	4.5	48	2.6%	-5.00 [-7.08, -2.92]
Liu 2017	14.13	4.2	120	17.75	5.23	120	3.5%	-3.62 [-4.82, -2.42]
Liu 2019	5.45	1.31	48	9.83	2.21	48	4.0%	-4.38 [-5.11, -3.65]
Ping 2022	4.65	1.2	46	7.91	1.74	46	4.0%	-3.26 [-3.87, -2.65]
Wang 2020	13.57	3.16	53	16.39	3.27	53	3.5%	-2.82 [-4.04, -1.60]
Zhao 2022	13.88	3.15	90	15.01	3.29	90	3.8%	-1.13 [-2.07, -0.19]
Zhong 2017	9.46	3.45	42	10.24	4.08	42	3.1%	-0.78 [-2.40, 0.84]
Zhu 2016	5.02	4.3	60	13.55	5.7	60	2.9%	-8.53 [-10.34, -6.72]
**Subtotal (95% CI)**			**643**			**639**	**39.0%**	**-3.10 [-4.03, -2.18]**
Heterogeneity: Tau² = 2.07; Chi² = 130.95, df = 10 (P < 0.00001); I² = 92%								
Test for overall effect: Z = 6.59 (P < 0.00001)								
**Total (95% CI)**			**1695**			**1690**	**100.0%**	**-3.39 [-3.94, -2.84]**
Heterogeneity: Tau² = 1.83; Chi² = 461.25, df = 28 (P < 0.00001); I² = 94%								
Test for overall effect: Z = 12.13 (P < 0.00001)								
Test for subgroup differences: Chi² = 0.63, df = 1 (P = 0.43), I² = 0%								

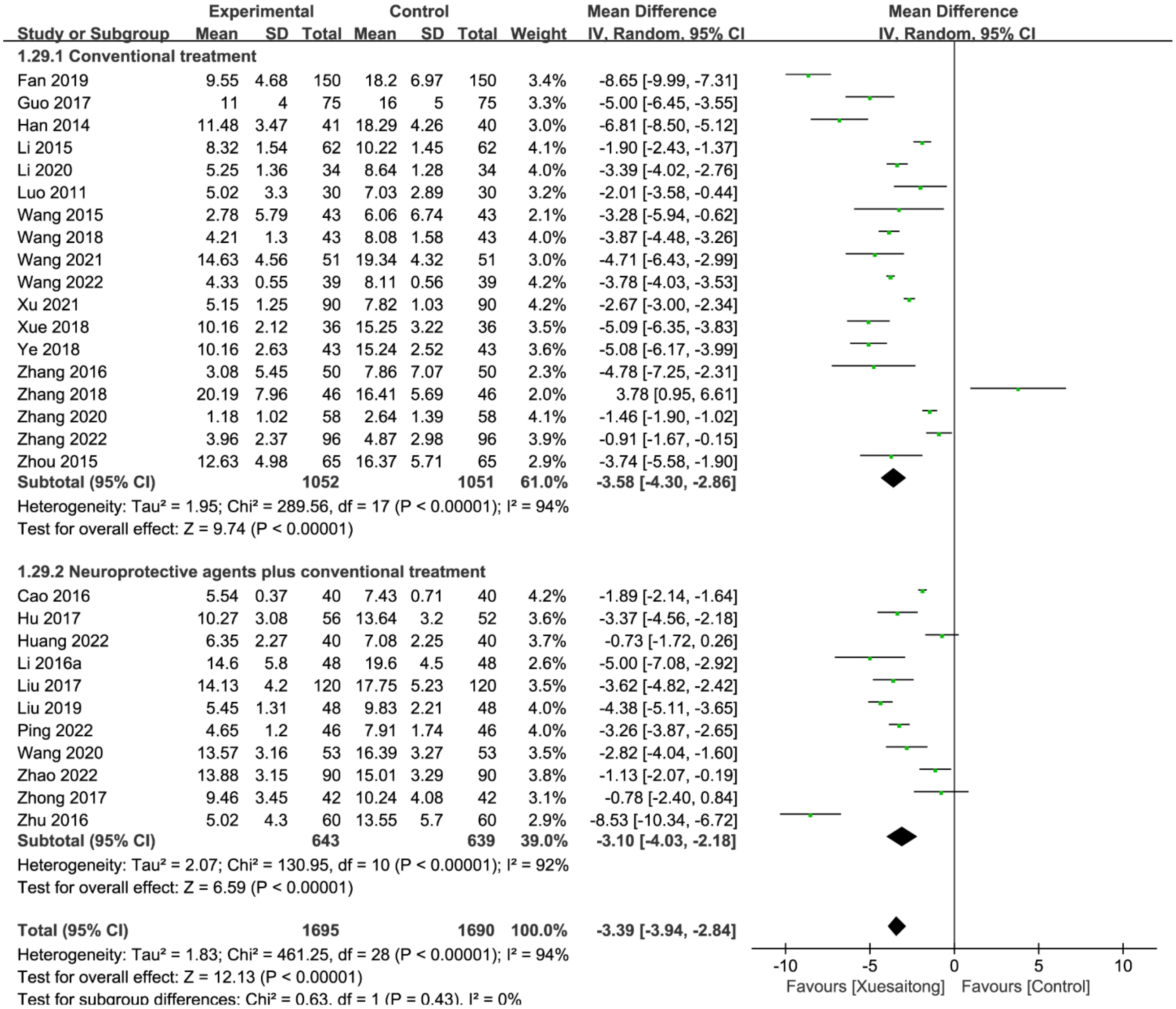

Supplementary Figure 8. Forest plot for the effect of Xuesaitong on European Stroke Scale score.

	Experimental			Control				Mean Difference
Study or Subgroup	Mean	SD	Total	Mean	SD	Total	Weight	IV, Random, 95% CI
Deng 2018	76.03	18.68	25	64.18	16.54	25	100.0%	11.85 [2.07, 21.63]
**Total (95% CI)**			**25**			**25**	**100.0%**	**11.85 [2.07, 21.63]**
Heterogeneity: Not applicable								
Test for overall effect: Z = 2.37 (P = 0.02)								

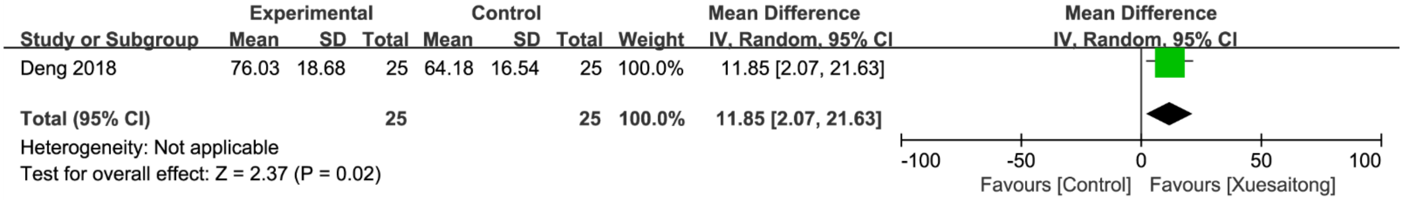

Supplementary Figure 9. Forest plot for the effect of Xuesaitong on total efficiency rate.

Study or Subgroup	Experimental Events	Experimental Total	Control Events	Control Total	Weight	Risk Ratio M-H, Random, 95% CI
Bu 2016	25	30	17	30	0.8%	1.47 [1.03, 2.09]
Cao 2016	38	40	36	40	3.2%	1.06 [0.93, 1.20]
Fan 2019	143	150	132	150	4.5%	1.08 [1.01, 1.16]
Fu 2011	60	64	49	58	3.2%	1.11 [0.98, 1.26]
Gao 2017	46	49	37	49	2.3%	1.24 [1.04, 1.48]
Guo 2017	73	75	65	75	3.9%	1.12 [1.02, 1.24]
Han 2014	38	41	33	40	2.4%	1.12 [0.95, 1.33]
Hu 2017	49	56	37	52	2.0%	1.23 [1.01, 1.50]
Huang 2016	52	62	60	62	3.4%	0.87 [0.77, 0.98]
Huang 2022	37	40	30	40	2.0%	1.23 [1.01, 1.51]
Li 2015	57	62	49	62	2.8%	1.16 [1.00, 1.35]
Li 2016a	45	48	39	48	2.6%	1.15 [0.99, 1.35]
Li 2016b	52	60	30	60	1.3%	1.73 [1.32, 2.27]
Li 2017	73	74	65	74	4.0%	1.12 [1.03, 1.23]
Li 2020	32	34	26	34	1.9%	1.23 [1.00, 1.51]
Liu 2017	113	120	89	120	3.4%	1.27 [1.13, 1.42]
Liu 2019	47	48	41	48	3.2%	1.15 [1.01, 1.30]
Luo 2011	28	30	21	30	1.4%	1.33 [1.04, 1.72]
Ou 2022	46	48	37	48	2.5%	1.24 [1.05, 1.47]
Song 2022	36	41	25	41	1.3%	1.44 [1.10, 1.89]
Wang 2015	41	43	32	43	2.1%	1.28 [1.06, 1.55]
Wang 2018	40	43	34	43	2.3%	1.18 [0.99, 1.40]
Wang 2020	50	53	42	53	2.7%	1.19 [1.02, 1.39]
Wang 2021	49	51	42	51	2.9%	1.17 [1.02, 1.34]
Wang 2022	32	39	24	39	1.2%	1.33 [1.00, 1.78]
Wu 2020	32	34	26	34	1.9%	1.23 [1.00, 1.51]
Xiao 2019	50	65	42	65	1.7%	1.19 [0.95, 1.49]
Xiao 2020	37	39	28	39	1.8%	1.32 [1.07, 1.63]
Xu 2021	87	90	78	90	4.0%	1.12 [1.02, 1.22]
Xue 2018	33	36	26	36	1.7%	1.27 [1.01, 1.59]
Yan 2023	49	51	42	51	2.9%	1.17 [1.02, 1.34]
Yang 2021	31	32	27	32	2.5%	1.15 [0.98, 1.35]
Zhang 2016	48	50	31	50	1.7%	1.55 [1.24, 1.94]
Zhang 2018	45	46	37	46	2.7%	1.22 [1.05, 1.41]
Zhang 2019	27	29	20	29	1.3%	1.35 [1.04, 1.76]
Zhang 2020	55	58	46	58	2.8%	1.20 [1.03, 1.38]
Zhang 2022	81	96	63	96	2.4%	1.29 [1.09, 1.52]
Zhao 2022	86	90	77	90	3.9%	1.12 [1.01, 1.23]
Zhou 2015	64	65	57	65	3.9%	1.12 [1.02, 1.24]
Zhu 2016	52	60	33	60	1.4%	1.58 [1.23, 2.02]
**Total (95% CI)**		**2242**		**2231**	**100.0%**	**1.19 [1.15, 1.23]**
Total events	2079		1725			

Heterogeneity: Tau² = 0.01; Chi² = 80.69, df = 39 (P < 0.0001); I² = 52%

Test for overall effect: Z = 9.61 (P < 0.00001)

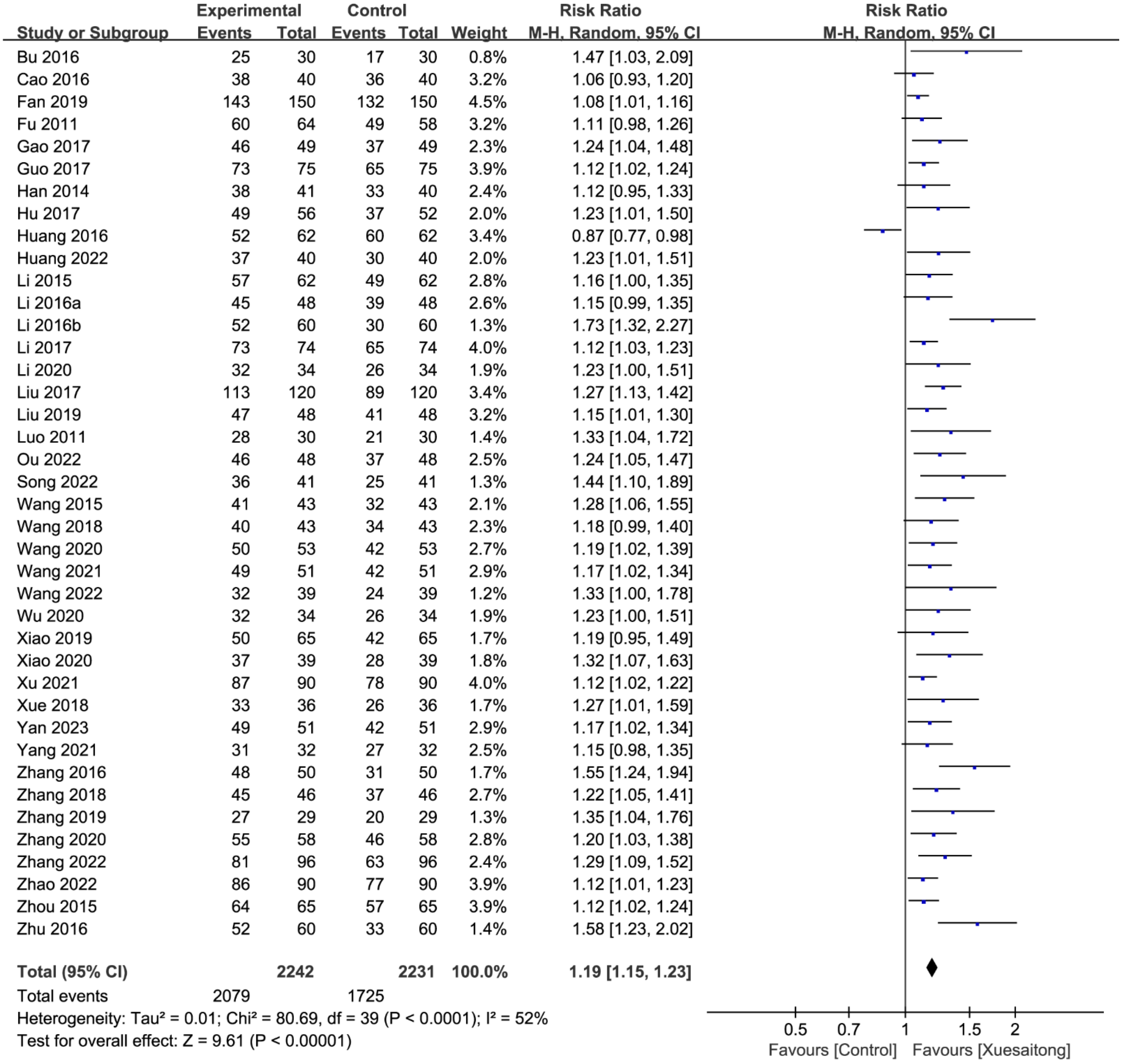

Supplementary Figure 10. Forest plot sensitivity analysis for the effect of Xuesaitong on total efficiency rate.

	Experimental		Control			Risk Ratio
Study or Subgroup	Events	Total	Events	Total	Weight	M-H, Random, 95% CI
Bu 2016	25	30	17	30	0.7%	1.47 [1.03, 2.09]
Cao 2016	38	40	36	40	3.5%	1.06 [0.93, 1.20]
Fan 2019	143	150	132	150	6.0%	1.08 [1.01, 1.16]
Fu 2011	60	64	49	58	3.4%	1.11 [0.98, 1.26]
Gao 2017	46	49	37	49	2.2%	1.24 [1.04, 1.48]
Guo 2017	73	75	65	75	4.6%	1.12 [1.02, 1.24]
Han 2014	38	41	33	40	2.4%	1.12 [0.95, 1.33]
Hu 2017	49	56	37	52	1.8%	1.23 [1.01, 1.50]
Huang 2016	52	62	60	62	0.0%	0.87 [0.77, 0.98]
Huang 2022	37	40	30	40	1.8%	1.23 [1.01, 1.51]
Li 2015	57	62	49	62	2.8%	1.16 [1.00, 1.35]
Li 2016a	45	48	39	48	2.6%	1.15 [0.99, 1.35]
Li 2016b	52	60	30	60	1.1%	1.73 [1.32, 2.27]
Li 2017	73	74	65	74	5.0%	1.12 [1.03, 1.23]
Li 2020	32	34	26	34	1.7%	1.23 [1.00, 1.51]
Liu 2017	113	120	89	120	3.8%	1.27 [1.13, 1.42]
Liu 2019	47	48	41	48	3.5%	1.15 [1.01, 1.30]
Luo 2011	28	30	21	30	1.2%	1.33 [1.04, 1.72]
Ou 2022	46	48	37	48	2.4%	1.24 [1.05, 1.47]
Song 2022	36	41	25	41	1.1%	1.44 [1.10, 1.89]
Wang 2015	41	43	32	43	2.0%	1.28 [1.06, 1.55]
Wang 2018	40	43	34	43	2.2%	1.18 [0.99, 1.40]
Wang 2020	50	53	42	53	2.7%	1.19 [1.02, 1.39]
Wang 2021	49	51	42	51	3.0%	1.17 [1.02, 1.34]
Wang 2022	32	39	24	39	1.0%	1.33 [1.00, 1.78]
Wu 2020	32	34	26	34	1.7%	1.23 [1.00, 1.51]
Xiao 2019	50	65	42	65	1.5%	1.19 [0.95, 1.49]
Xiao 2020	37	39	28	39	1.6%	1.32 [1.07, 1.63]
Xu 2021	87	90	78	90	4.9%	1.12 [1.02, 1.22]
Xue 2018	33	36	26	36	1.5%	1.27 [1.01, 1.59]
Yan 2023	49	51	42	51	3.0%	1.17 [1.02, 1.34]
Yang 2021	31	32	27	32	2.5%	1.15 [0.98, 1.35]
Zhang 2016	48	50	31	50	1.5%	1.55 [1.24, 1.94]
Zhang 2018	45	46	37	46	2.8%	1.22 [1.05, 1.41]
Zhang 2019	27	29	20	29	1.1%	1.35 [1.04, 1.76]
Zhang 2020	55	58	46	58	2.9%	1.20 [1.03, 1.38]
Zhang 2022	81	96	63	96	2.3%	1.29 [1.09, 1.52]
Zhao 2022	86	90	77	90	4.6%	1.12 [1.01, 1.23]
Zhou 2015	64	65	57	65	4.6%	1.12 [1.02, 1.24]
Zhu 2016	52	60	33	60	1.2%	1.58 [1.23, 2.02]
**Total (95% CI)**		**2180**		**2169**	**100.0%**	**1.19 [1.15, 1.22]**
Total events	2027		1665			

Heterogeneity: Tau² = 0.00; Chi² = 55.98, df = 38 (P = 0.03); I² = 32%

Test for overall effect: Z = 11.40 (P < 0.00001)

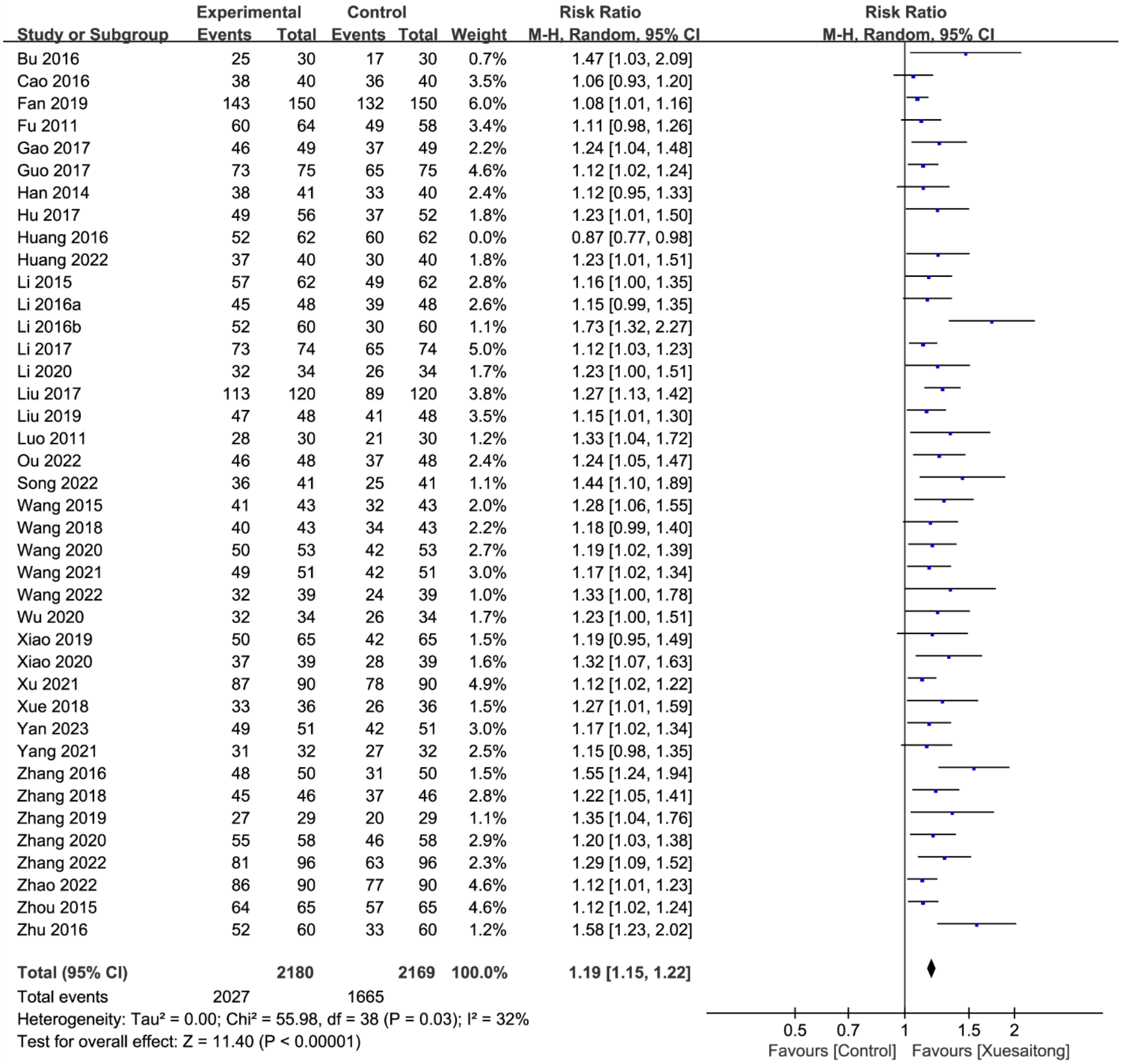

Supplementary Figure 11. Forest plot of subgroup analysis by duration of treatment for the effect of Xuesaitong on total efficiency rate.

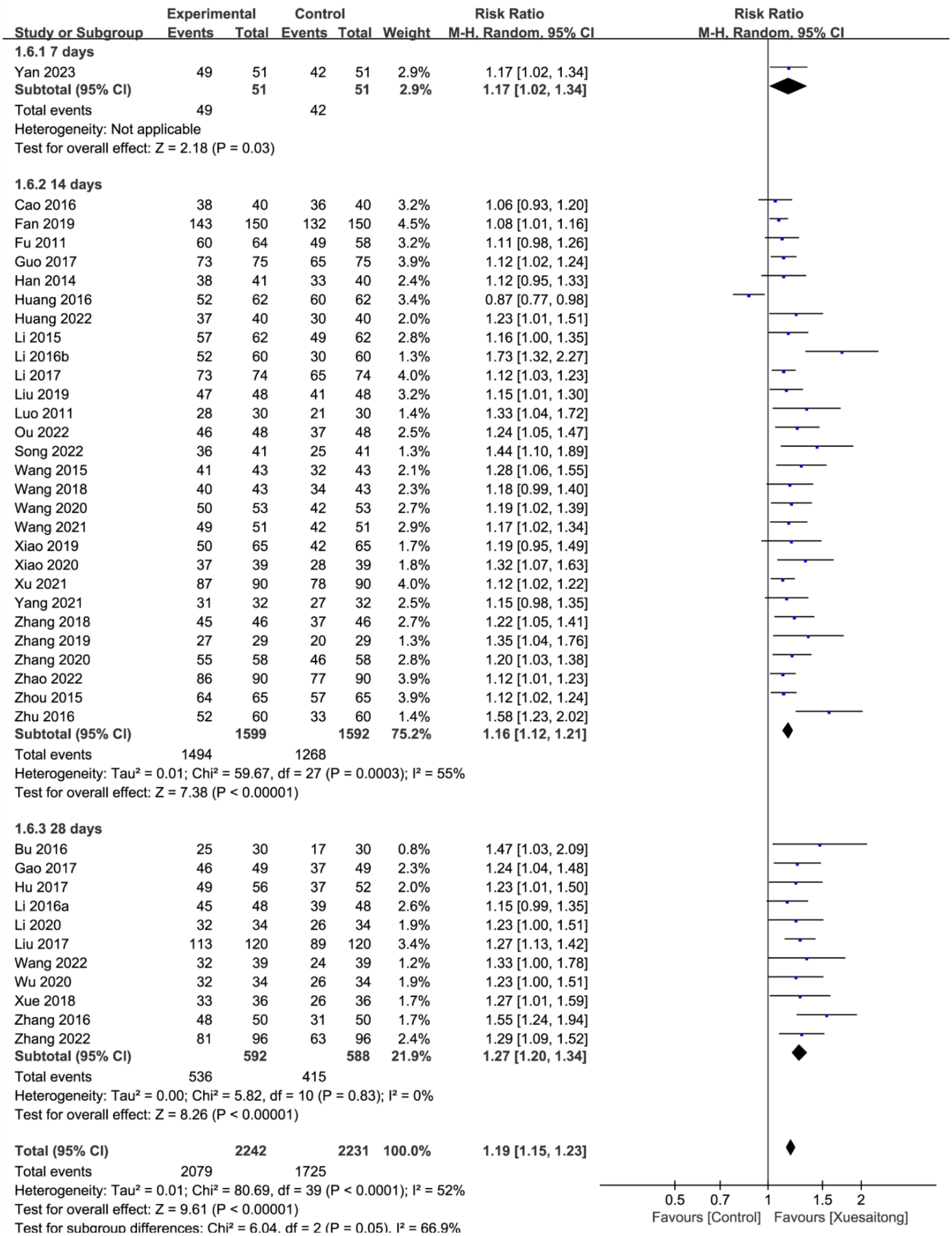

Supplementary Figure 12. Forest plot of subgroup analysis by dosage form for the effect of Xuesaitong on total efficiency rate.

Study or Subgroup	Experimental Events	Experimental Total	Control Events	Control Total	Weight	Risk Ratio M-H, Random, 95% CI
**1.7.1 Xuesaitong injection**						
Bu 2016	25	30	17	30	0.8%	1.47 [1.03, 2.09]
Cao 2016	38	40	36	40	3.2%	1.06 [0.93, 1.20]
Fan 2019	143	150	132	150	4.5%	1.08 [1.01, 1.16]
Fu 2011	60	64	49	58	3.2%	1.11 [0.98, 1.26]
Gao 2017	46	49	37	49	2.3%	1.24 [1.04, 1.48]
Guo 2017	73	75	65	75	3.9%	1.12 [1.02, 1.24]
Han 2014	38	41	33	40	2.4%	1.12 [0.95, 1.33]
Hu 2017	49	56	37	52	2.0%	1.23 [1.01, 1.50]
Huang 2016	52	62	60	62	3.4%	0.87 [0.77, 0.98]
Huang 2022	37	40	30	40	2.0%	1.23 [1.01, 1.51]
Li 2015	57	62	49	62	2.8%	1.16 [1.00, 1.35]
Li 2016a	45	48	39	48	2.6%	1.15 [0.99, 1.35]
Li 2016b	52	60	30	60	1.3%	1.73 [1.32, 2.27]
Li 2017	73	74	65	74	4.0%	1.12 [1.03, 1.23]
Li 2020	32	34	26	34	1.9%	1.23 [1.00, 1.51]
Liu 2017	113	120	89	120	3.4%	1.27 [1.13, 1.42]
Liu 2019	47	48	41	48	3.2%	1.15 [1.01, 1.30]
Luo 2011	28	30	21	30	1.4%	1.33 [1.04, 1.72]
Ou 2022	46	48	37	48	2.5%	1.24 [1.05, 1.47]
Song 2022	36	41	25	41	1.3%	1.44 [1.10, 1.89]
Wang 2015	41	43	32	43	2.1%	1.28 [1.06, 1.55]
Wang 2018	40	43	34	43	2.3%	1.18 [0.99, 1.40]
Wang 2020	50	53	42	53	2.7%	1.19 [1.02, 1.39]
Wang 2021	49	51	42	51	2.9%	1.17 [1.02, 1.34]
Wang 2022	32	39	24	39	1.2%	1.33 [1.00, 1.78]
Wu 2020	32	34	26	34	1.9%	1.23 [1.00, 1.51]
Xiao 2019	50	65	42	65	1.7%	1.19 [0.95, 1.49]
Xiao 2020	37	39	28	39	1.8%	1.32 [1.07, 1.63]
Xu 2021	87	90	78	90	4.0%	1.12 [1.02, 1.22]
Xue 2018	33	36	26	36	1.7%	1.27 [1.01, 1.59]
Yan 2023	49	51	42	51	2.9%	1.17 [1.02, 1.34]
Yang 2021	31	32	27	32	2.5%	1.15 [0.98, 1.35]
Zhang 2018	45	46	37	46	2.7%	1.22 [1.05, 1.41]
Zhang 2019	27	29	20	29	1.3%	1.35 [1.04, 1.76]
Zhang 2020	55	58	46	58	2.8%	1.20 [1.03, 1.38]
Zhang 2022	81	96	63	96	2.4%	1.29 [1.09, 1.52]
Zhao 2022	86	90	77	90	3.9%	1.12 [1.01, 1.23]
Zhou 2015	64	65	57	65	3.9%	1.12 [1.02, 1.24]
Zhu 2016	52	60	33	60	1.4%	1.58 [1.23, 2.02]
**Subtotal (95% CI)**		**2192**		**2181**	**98.3%**	**1.18 [1.14, 1.22]**
Total events	2031		1694			
Heterogeneity: Tau² = 0.00; Chi² = 72.84, df = 38 (P = 0.0006); I² = 48%						
Test for overall effect: Z = 9.56 (P < 0.00001)						
**1.7.2 Xuesaitong oral preparation**						
Zhang 2016	48	50	31	50	1.7%	1.55 [1.24, 1.94]
**Subtotal (95% CI)**		**50**		**50**	**1.7%**	**1.55 [1.24, 1.94]**
Total events	48		31			
Heterogeneity: Not applicable						
Test for overall effect: Z = 3.82 (P = 0.0001)						
**Total (95% CI)**		**2242**		**2231**	**100.0%**	**1.19 [1.15, 1.23]**
Total events	2079		1725			
Heterogeneity: Tau² = 0.01; Chi² = 80.69, df = 39 (P < 0.0001); I² = 52%						
Test for overall effect: Z = 9.61 (P < 0.00001)						
Test for subgroup differences: Chi² = 5.56, df = 1 (P = 0.02), I² = 82.0%						

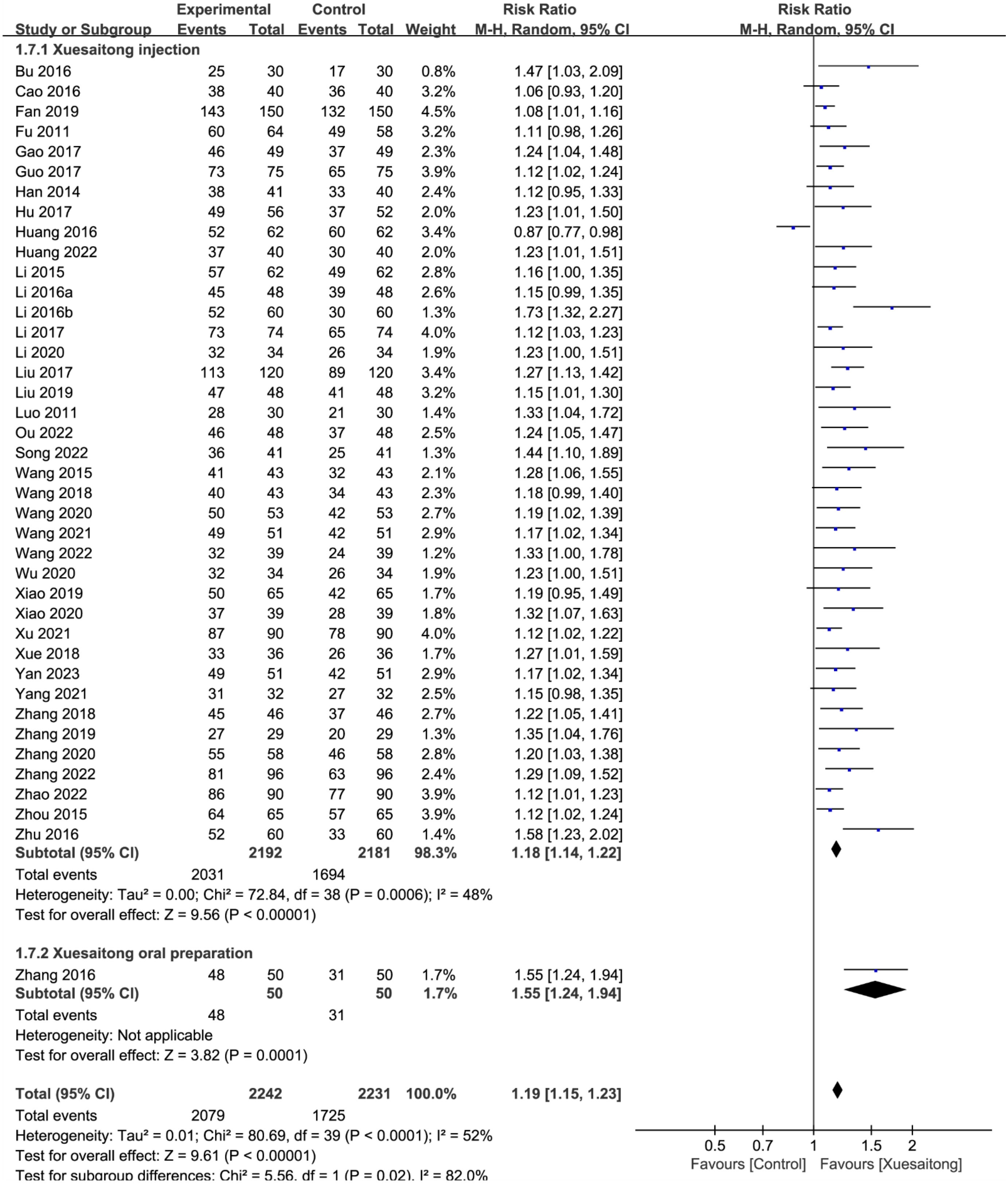

Supplementary Figure 13. Forest plot of subgroup analysis by the combination treatment for the effect of Xuesaitong on total efficiency rate.

Study or Subgroup	Experimental Events	Experimental Total	Control Events	Control Total	Weight	Risk Ratio M-H, Random, 95% CI
**1.27.1 Conventional treatment**						
Fan 2019	143	150	132	150	4.5%	1.08 [1.01, 1.16]
Fu 2011	60	64	49	58	3.2%	1.11 [0.98, 1.26]
Guo 2017	73	75	65	75	3.9%	1.12 [1.02, 1.24]
Han 2014	38	41	33	40	2.4%	1.12 [0.95, 1.33]
Huang 2016	52	62	60	62	3.4%	0.87 [0.77, 0.98]
Li 2015	57	62	49	62	2.8%	1.16 [1.00, 1.35]
Li 2016b	52	60	30	60	1.3%	1.73 [1.32, 2.27]
Li 2020	32	34	26	34	1.9%	1.23 [1.00, 1.51]
Luo 2011	28	30	21	30	1.4%	1.33 [1.04, 1.72]
Wang 2015	41	43	32	43	2.1%	1.28 [1.06, 1.55]
Wang 2018	40	43	34	43	2.3%	1.18 [0.99, 1.40]
Wang 2021	49	51	42	51	2.9%	1.17 [1.02, 1.34]
Wang 2022	32	39	24	39	1.2%	1.33 [1.00, 1.78]
Xiao 2019	50	65	42	65	1.7%	1.19 [0.95, 1.49]
Xiao 2020	37	39	28	39	1.8%	1.32 [1.07, 1.63]
Xu 2021	87	90	78	90	4.0%	1.12 [1.02, 1.22]
Xue 2018	33	36	26	36	1.7%	1.27 [1.01, 1.59]
Yan 2023	49	51	42	51	2.9%	1.17 [1.02, 1.34]
Zhang 2016	48	50	31	50	1.7%	1.55 [1.24, 1.94]
Zhang 2018	45	46	37	46	2.7%	1.22 [1.05, 1.41]
Zhang 2019	27	29	20	29	1.3%	1.35 [1.04, 1.76]
Zhang 2020	55	58	46	58	2.8%	1.20 [1.03, 1.38]
Zhang 2022	81	96	63	96	2.4%	1.29 [1.09, 1.52]
Zhou 2015	64	65	57	65	3.9%	1.12 [1.02, 1.24]
**Subtotal (95% CI)**		**1379**		**1372**	**60.2%**	**1.18 [1.13, 1.24]**
Total events	1273		1067			
Heterogeneity: Tau² = 0.01; Chi² = 58.25, df = 23 (P < 0.0001); I² = 61%						
Test for overall effect: Z = 6.69 (P < 0.00001)						
**1.27.2 Neuroprotective agents plus conventional treatment**						
Bu 2016	25	30	17	30	0.8%	1.47 [1.03, 2.09]
Cao 2016	38	40	36	40	3.2%	1.06 [0.93, 1.20]
Gao 2017	46	49	37	49	2.3%	1.24 [1.04, 1.48]
Hu 2017	49	56	37	52	2.0%	1.23 [1.01, 1.50]
Huang 2022	37	40	30	40	2.0%	1.23 [1.01, 1.51]
Li 2016a	45	48	39	48	2.6%	1.15 [0.99, 1.35]
Li 2017	73	74	65	74	4.0%	1.12 [1.03, 1.23]
Liu 2017	113	120	89	120	3.4%	1.27 [1.13, 1.42]
Liu 2019	47	48	41	48	3.2%	1.15 [1.01, 1.30]
Ou 2022	46	48	37	48	2.5%	1.24 [1.05, 1.47]
Song 2022	36	41	25	41	1.3%	1.44 [1.10, 1.89]
Wang 2020	50	53	42	53	2.7%	1.19 [1.02, 1.39]
Wu 2020	32	34	26	34	1.9%	1.23 [1.00, 1.51]
Yang 2021	31	32	27	32	2.5%	1.15 [0.98, 1.35]
Zhao 2022	86	90	77	90	3.9%	1.12 [1.01, 1.23]
Zhu 2016	52	60	33	60	1.4%	1.58 [1.23, 2.02]
**Subtotal (95% CI)**		**863**		**859**	**39.8%**	**1.19 [1.14, 1.25]**
Total events	806		658			
Heterogeneity: Tau² = 0.00; Chi² = 20.92, df = 15 (P = 0.14); I² = 28%						
Test for overall effect: Z = 7.47 (P < 0.00001)						
**Total (95% CI)**		**2242**		**2231**	**100.0%**	**1.19 [1.15, 1.23]**
Total events	2079		1725			
Heterogeneity: Tau² = 0.01; Chi² = 80.69, df = 39 (P < 0.0001); I² = 52%						
Test for overall effect: Z = 9.61 (P < 0.00001)						
Test for subgroup differences: Chi² = 0.02, df = 1 (P = 0.88), I² = 0%						

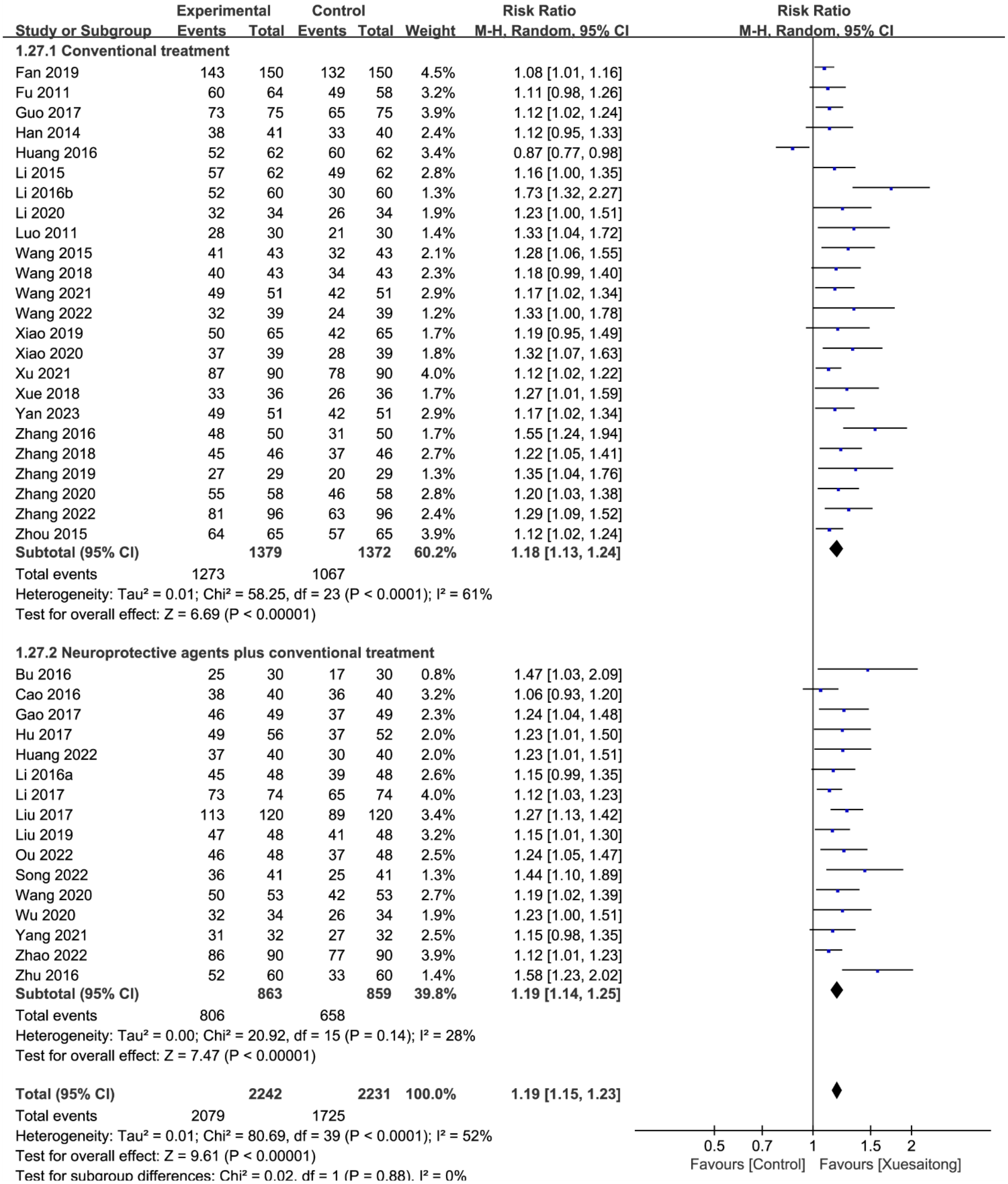

Supplementary Figure 14. Forest plot of subgroup analysis by the time of administration for the effect of Xuesaitong on total efficiency rate.

Study or Subgroup	Experimental Events	Experimental Total	Control Events	Control Total	Weight	Risk Ratio M-H, Random, 95% CI
**1.26.1 Treatment initiated within 72 hours**						
Bu 2016	25	30	17	30	0.8%	1.47 [1.03, 2.09]
Cao 2016	38	40	36	40	3.2%	1.06 [0.93, 1.20]
Fan 2019	143	150	132	150	4.5%	1.08 [1.01, 1.16]
Fu 2011	60	64	49	58	3.2%	1.11 [0.98, 1.26]
Gao 2017	46	49	37	49	2.3%	1.24 [1.04, 1.48]
Guo 2017	73	75	65	75	3.9%	1.12 [1.02, 1.24]
Han 2014	38	41	33	40	2.4%	1.12 [0.95, 1.33]
Hu 2017	49	56	37	52	2.0%	1.23 [1.01, 1.50]
Huang 2016	52	62	60	62	3.4%	0.87 [0.77, 0.98]
Huang 2022	37	40	30	40	2.0%	1.23 [1.01, 1.51]
Li 2015	57	62	49	62	2.8%	1.16 [1.00, 1.35]
Li 2016a	45	48	39	48	2.6%	1.15 [0.99, 1.35]
Li 2016b	52	60	30	60	1.3%	1.73 [1.32, 2.27]
Li 2020	32	34	26	34	1.9%	1.23 [1.00, 1.51]
Liu 2017	113	120	89	120	3.4%	1.27 [1.13, 1.42]
Liu 2019	47	48	41	48	3.2%	1.15 [1.01, 1.30]
Luo 2011	28	30	21	30	1.4%	1.33 [1.04, 1.72]
Ou 2022	46	48	37	48	2.5%	1.24 [1.05, 1.47]
Song 2022	36	41	25	41	1.3%	1.44 [1.10, 1.89]
Wang 2015	41	43	32	43	2.1%	1.28 [1.06, 1.55]
Wang 2018	40	43	34	43	2.3%	1.18 [0.99, 1.40]
Wang 2020	50	53	42	53	2.7%	1.19 [1.02, 1.39]
Wang 2021	49	51	42	51	2.9%	1.17 [1.02, 1.34]
Wang 2022	32	39	24	39	1.2%	1.33 [1.00, 1.78]
Xiao 2019	50	65	42	65	1.7%	1.19 [0.95, 1.49]
Xiao 2020	37	39	28	39	1.8%	1.32 [1.07, 1.63]
Xu 2021	87	90	78	90	4.0%	1.12 [1.02, 1.22]
Xue 2018	33	36	26	36	1.7%	1.27 [1.01, 1.59]
Yan 2023	49	51	42	51	2.9%	1.17 [1.02, 1.34]
Yang 2021	31	32	27	32	2.5%	1.15 [0.98, 1.35]
Zhang 2016	48	50	31	50	1.7%	1.55 [1.24, 1.94]
Zhang 2018	45	46	37	46	2.7%	1.22 [1.05, 1.41]
Zhang 2019	27	29	20	29	1.3%	1.35 [1.04, 1.76]
Zhang 2020	55	58	46	58	2.8%	1.20 [1.03, 1.38]
Zhang 2022	81	96	63	96	2.4%	1.29 [1.09, 1.52]
Zhao 2022	86	90	77	90	3.9%	1.12 [1.01, 1.23]
Zhou 2015	64	65	57	65	3.9%	1.12 [1.02, 1.24]
Zhu 2016	52	60	33	60	1.4%	1.58 [1.23, 2.02]
**Subtotal (95% CI)**		**2134**		**2123**	**94.1%**	**1.19 [1.15, 1.23]**
Total events	1974		1634			
Heterogeneity: Tau² = 0.01; Chi² = 79.57, df = 37 (P < 0.0001); I² = 54%						
Test for overall effect: Z = 9.22 (P < 0.00001)						
**1.26.2 Treatment initiated within 14 days (except for studies initiated within 72 hours only)**						
Li 2017	73	74	65	74	4.0%	1.12 [1.03, 1.23]
Wu 2020	32	34	26	34	1.9%	1.23 [1.00, 1.51]
**Subtotal (95% CI)**		**108**		**108**	**5.9%**	**1.14 [1.05, 1.24]**
Total events	105		91			
Heterogeneity: Tau² = 0.00; Chi² = 0.74, df = 1 (P = 0.39); I² = 0%						
Test for overall effect: Z = 3.14 (P = 0.002)						
**Total (95% CI)**		**2242**		**2231**	**100.0%**	**1.19 [1.15, 1.23]**
Total events	2079		1725			
Heterogeneity: Tau² = 0.01; Chi² = 80.69, df = 39 (P < 0.0001); I² = 52%						
Test for overall effect: Z = 9.61 (P < 0.00001)						
Test for subgroup differences: Chi² = 0.89, df = 1 (P = 0.35), I² = 0%						

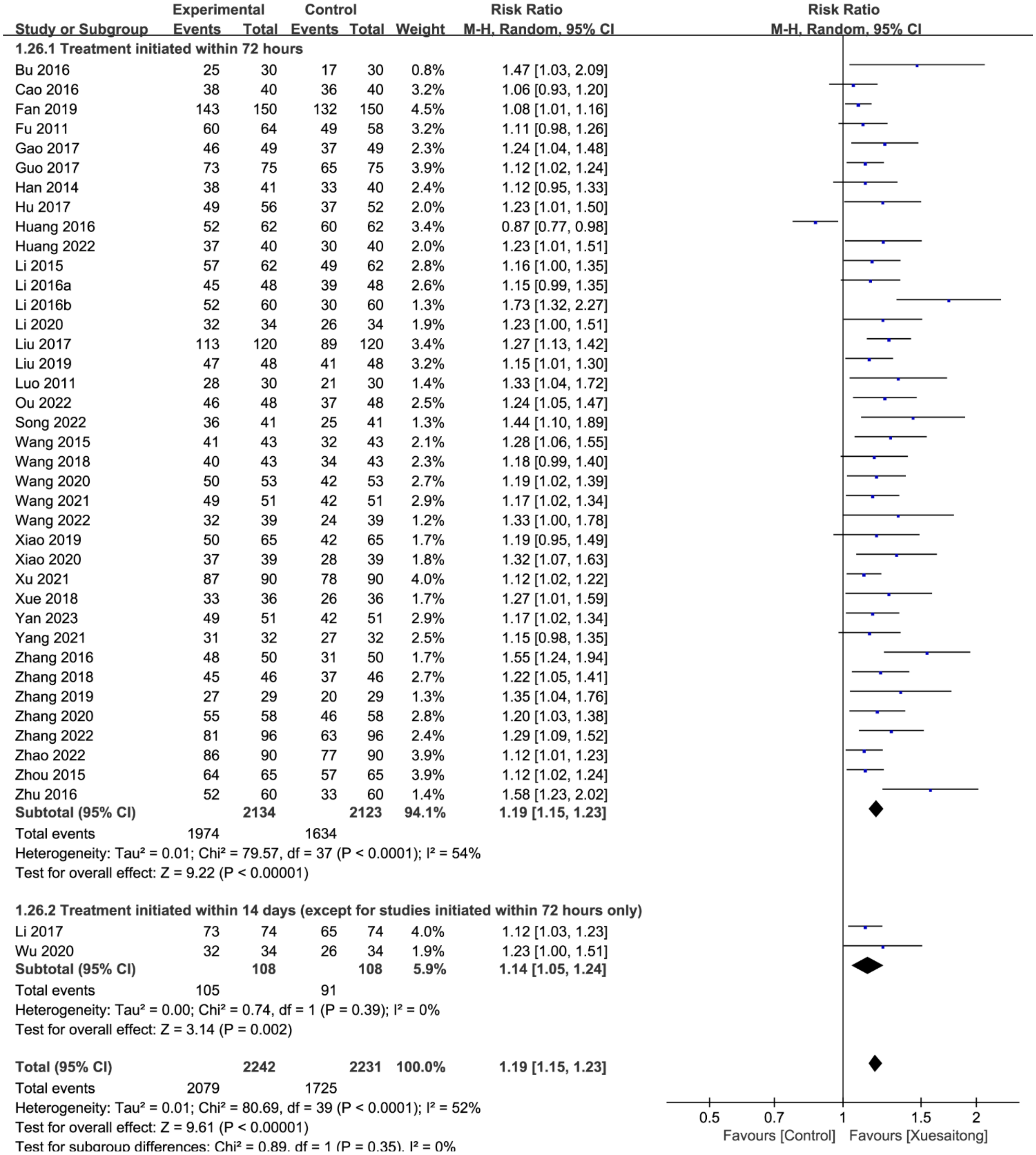

Supplementary Figure 15. Forest plot for the effect of Xuesaitong on (A) Whole blood high-cut viscosity, (B) Whole blood low-cut viscosity, (C) Fibrinogen, (D) Plasma viscosity, (E) Hematocrit.

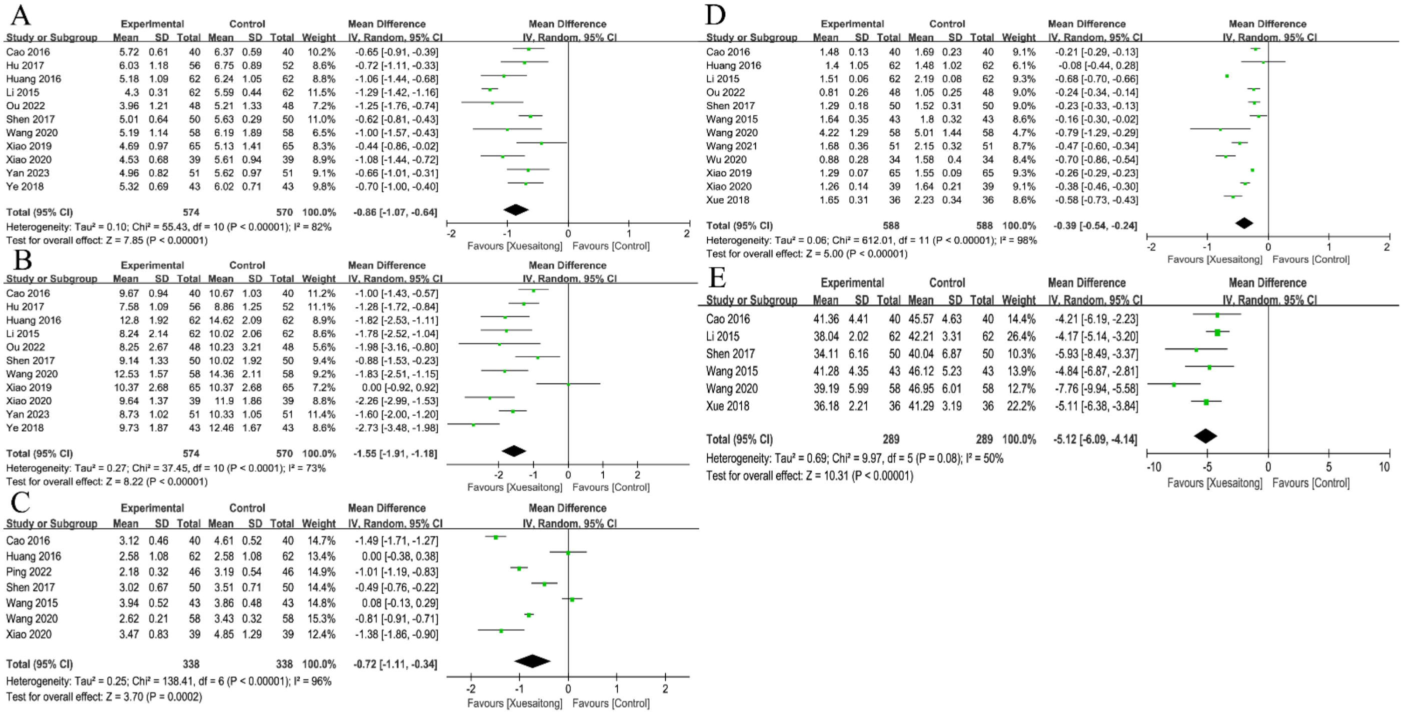

Supplementary Figure 16. Funnel plots of (A) Barthel Index score, (B) National Institute of Health Stroke Scale score, (C) Total efficiency rate, (D) Whole blood high-cut viscosity, (E) Whole blood low-cut viscosity, (F) Plasma viscosity, (G) Adverse events.

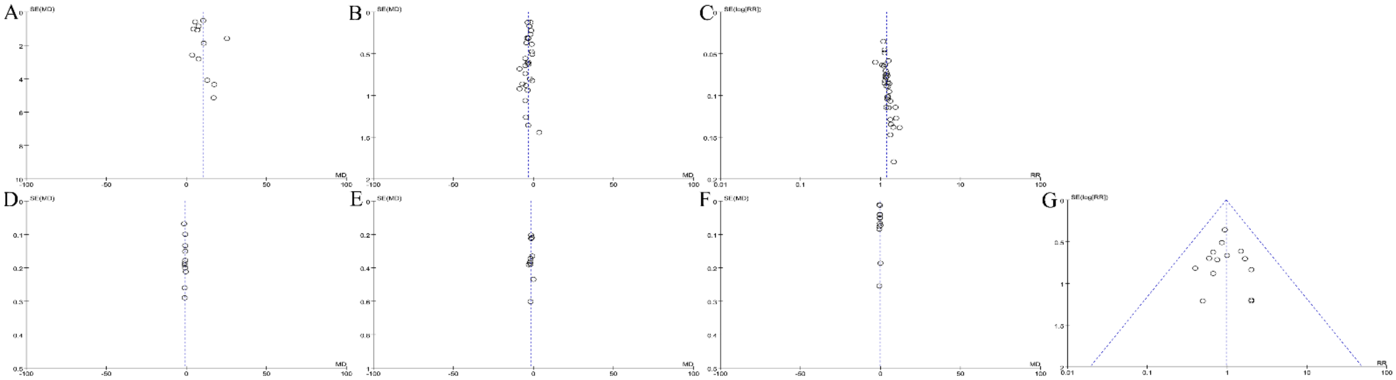

Supplement: Supplementary file 1 [file DataSheet1.pdf]
